# Reproductive and immune-related consequences of *Achillea fragrantissima* (Forssk.) in male rats via modulation of steroidogenesis- and immune-related genes expression

**DOI:** 10.3389/fvets.2026.1746307

**Published:** 2026-04-16

**Authors:** Ehsan H. Abu-Zeid, Hassan M. Emam, Noura A. Abd-Allah, Mohammed S. Sobh, Tarek Khamis, Reham H. El-Attar, Shereen El. Abdel-Hamid, Ibrahim F. Rehan, Asmaa Elnagar, František Zigo, Martina Zigová, Esraa M. Fahmy

**Affiliations:** 1 Department of Forensic Medicine and Toxicology, Faculty of Veterinary Medicine, Zagazig University, Zagazig, Egypt; 2 Department of Anatomy and Embryology, Faculty of Veterinary Medicine, Zagazig University, Zagazig, Egypt; 3 Department of Clinical Pathology, Faculty of Veterinary Medicine, Zagazig University, Zagazig, Egypt; 4 Department of Pathology, Faculty of Veterinary Medicine, Zagazig University, Zagazig, Egypt; 5 Department of Pharmacology, Faculty of Veterinary Medicine, Zagazig University, Zagazig, Egypt; 6 General Administration for Medical Affairs, Zagazig University, Zagazig, Egypt; 7 Department of Behavior and Management of Animal, Poultry, and Aquatics, Faculty of Veterinary Medicine, Zagazig University, Zagazig, Egypt; 8 Department of Animal Behaviour and Husbandry, Faculty of Veterinary Medicine, Menoufia University, Shebin Alkom, Menoufia, Egypt; 9 Institute of Genetics and Animal Biotechnology of the Polish Academy of Sciences, Jastrzębiec, Poland; 10 Department of Nutrition and Animal Husbandry, University of Veterinary Medicine and Pharmacy, Košice, Slovakia; 11 Department of Pharmacology, Faculty of Medicine, Pavol Jozef Šafárik University, Košice, Slovakia

**Keywords:** *Achillea fragrantissima*, *Caspase*-3, *CYP11A1*, *HSD17B3*, male reproductive performance, oxidative stress, phagocytosis assay, StAR

## Abstract

**Introduction:**

*Achillea fragrantissima* (AFG) is known for its medicinal properties, while different Achillea species have shown both beneficial and detrimental impacts on male fertility. This investigation evaluated the effects of AFG oral administration on behavior, steroidogenesis, and immune-related gene expression in Sprague-Dawley male rats.

**Methods:**

Forty-five mature rats were randomly grouped into: Control, Low-dose group (AFG-L: 500 mg/kg extract), and High-dose group (AFG-H: 1000 mg/kg extract). The AFG extract was orally administered for 30 days. Behavioral, hematological, reproductive, immune, and oxidative stress-related indices were evaluated.

**Results:**

The AFG administration at both doses significantly (*p* < 0.05) modulated the feeding and drinking, hole-board, swimming performance, tube dominance, and modified Y-maze tests outcomes. The AFG-L group displayed significant (*p* < 0.05) increases in final weight gain, sperm count, motility, and viable sperms %, with significant (*p* < 0.05) reductions in aberrant and dead sperms %. AFG significantly (*p* < 0.05) increased phagocytic index (PhI) and phagocytic %. However, erythrogram and leukocyte counts were not significantly altered. The AFG-L group displayed significant (*p* < 0.05) increases in serum testosterone (TEST), FSH, LH, and Estradiol (E2) hormones. Additionally, interleukin-6 (IL-6) and tumor necrosis factor-alpha (TNF-ɑ) concentrations were significantly decreased (*p* < 0.05). AFG extract administration significantly improved (*p* < 0.05) lipid profile, increased the testicular and splenic glutathione peroxidase (GPx), superoxide dismutase (SOD), total antioxidant capacity (TAC) levels, whereas the malondialdehyde (MDA), protein carbonyl (PCO), 8-hydroxy-2-deoxyguanosine (8OH2dG), and testicular lactate dehydrogenase (LDH) levels were significantly reduced (*p* < 0.05). AFG administration significantly (*p* < 0.05) upregulated mRNA expression of *StAR, CYP11A1, CYP17A1*, and *HSD17B3* in the testes, as well as *CD3, CD4, CD20*, and *IL-10* in the spleen, but expression levels of *CYP19A1* and *Caspase*-3 in testes, along with CD8 and Caspase-3 in spleen, were significantly (*p* < 0.05) downregulated. The AFG-L group maintained normal testicular architecture with preserved seminiferous tubules and only mild interstitial edema alongside mild splenic sinusoid dilatation with macrophage infiltration. In contrast, the AFG-H group exhibited significant histopathological alterations, including irregular seminiferous tubules, reduced mature sperm, and increased interstitial edema, while the spleen showed preserved lymphoid follicles with congested vasculature.

**Discussion:**

The findings of the ongoing work demonstrate for the first time that *A. fragrantissima* extract exerts reproductive and immune-enhancing outcomes in male rats, with the low dose exhibiting greater efficacy than the high dose, indicating a clear dose-dependent response.

## Graphical abstract

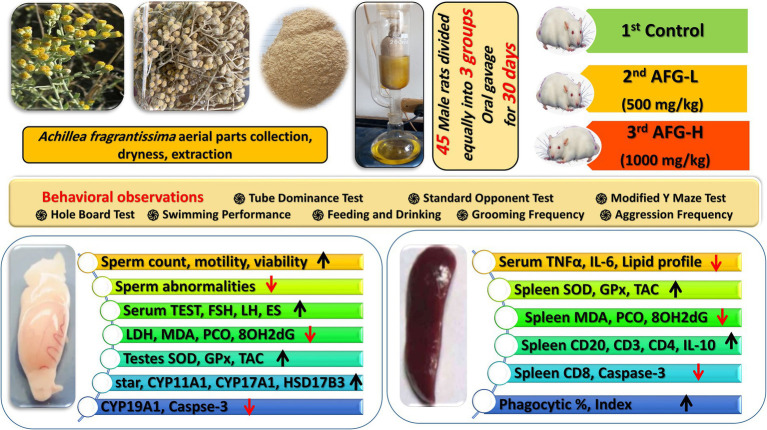

## 1 Introduction

Male infertility is a complex issue that profoundly affects personal lives and societal structures. It refers to the incapacity of an adult male to induce conception in a productive female after 1 year of consistent, unprotected coitus. Clinical criteria frequently classify male infertility according to aberrant semen parameters, including low sperm count (oligospermia), absence of sperm (azospermia), or suboptimal sperm motility and morphology (1).

Infertility persists as a significant public health concern, impacting around 186 million individuals worldwide, as reported by the World Health Organization (2). According to Roomaney et al. (3), around 8–12% of couples worldwide experience infertility, where most couples undergoing treatment for infertility issues reside in emerging nations, particularly those in central and western Africa. Irrespective of this fact, there is a dearth of research on infertility in developing countries (4).

The potential of plant extracts to increase animal reproductive efficiency has been investigated. These extracts contain several phytochemicals that have been shown to significantly alter reproductive parameters, including libido, fertility, and overall reproductive health (5). It is essential to optimize reproductive performance to maintain and improve livestock production. Herbal therapy presents a potential and culturally relevant method for enhancing male reproductive health, along with modern health views on preventive and holistic wellbeing. Additional research is needed to corroborate the findings and elucidate the mechanisms of action (6).

Throughout diverse cultural contexts, the use of botanical therapies in traditional medicine to enhance reproductive potential and promote fertility has been employed for centuries, garnering increased interest worldwide (7–12). Different phytoconstituents found in medicinal herbs have the potential to alleviate reproductive health issues. Phenolics, triterpenoids, flavonoids, cardiac glycosides, steroids, quinones, terpenoids, anthocyanins, and alkaloids are the main classes of phytochemicals (13, 14). Numerous discovered phytocompounds have been documented to enhance reproductive parameters, including motility, sperm count, and viability. These phytocompounds have been documented to augment indicators of oxidative stress (OS), such as glutathione peroxidase (GPx), SOD, and catalase (CAT) (15). It has been shown that they also experimentally enhance the overall histoarchitectures of the testes (12).

*Achillea fragrantissima*, a perennial herb commonly used in traditional medicine, is valued for its medicinal properties in treating gastrointestinal disorders, wounds, and infections (16–18). The pharmacological effects can be ascribed to the presence of a variety of bioactive compounds. The herb exhibits notable anti-inflammatory properties, which may be advantageous for chronic illnesses such as arthritis and other inflammatory conditions (19). Furthermore, its extracts demonstrate antibacterial activities, including efficacy against methicillin-resistant *Staphylococcus aureus*, and indicate potential in inhibiting viral infections such as influenza (18, 20). *Achillea fragrantissima* methanolic extract suppresses the H1N1 influenza virus (21). Phytochemicals, including essential oils, flavonoids, sesquiterpene lactones, and phenolic acids, are regarded as the primary constituents responsible for the therapeutic advantages of *A. fragrantissima* (22). These compounds are widely acknowledged for their anti-inflammatory, antibacterial, and antioxidant activities. Flavonoids control key cellular pathways related to inflammation and immunity, whereas sesquiterpene lactones have both anti-inflammatory and antibacterial effects (19). Furthermore, the potent antioxidant characteristics of *A. fragrantissima*, attributed to its high phenolic content, help safeguard against oxidative stress, a factor associated with chronic conditions such as cardiovascular diseases, cancer, and diabetes (18). Prior studies have examined the antiviral characteristics of the ethanolic extract of *A. fragrantissima*, which has shown efficacy against rotavirus, suggesting its potential application in antiviral therapies (23).

The impacts of different *Achillea* species on male fertility are controversial, with research indicating both possible adverse and beneficial effects. *A. millefolium* extract gavaged for 14 days at doses of 200 or 400 mg/kg, protected from paclitaxel-triggered testicular damage, and represents a favorable natural herb with the possibility to enhance male productiveness. *A. millefolium* enhanced the concentrations of testosterone (TEST), follicle-stimulating hormone (FSH), and luteinizing hormone (LH), elevated enzymatic antioxidants in testes, reduced inflammation, reinstated the histological architecture of the testis, and markedly diminished oxidative destruction of DNA as well as apoptotic changes via lowering *Caspase-3* expressions (24). *A. millefolium* at a dose of 120 mg/kg B.wt., daily over 48 days protected against male rat reproductive failure induced by nicotine exposure, reduced the aberrant and dead sperm, increased sperm count, motility, and tubule differentiation index, decreased LDH, total nitrite, MDA and increased serum FSH, LH, TEST, TAC, SOD, and total thiol molecules levels, which may be due to its antioxidant capability (25). Meanwhile, dosing of 200 and 400 mg/kg alcoholic *A. millefolium* extracts reduced fertility by lowering TEST levels, decreasing sperm motility and viability, and reducing the reproductive organs and final body weights (26). *A. millefolium* inflorescences aqueous extract at 1.2 g/kg gavaged for 28 days protected male Wistar rats against cyclophosphamide-prompted reproductive injury, showing significant recovery from reductions in the epididymides, testes, and body weights, along with histological alterations. Also, the extract improved the reduced epididymal sperm count, TEST, sperm activity, testicular antioxidant capacity, and stereological parameters (27).

Additionally, adult male Wistar rats gavaged or intraperitoneally injected with 200, 400, and 800 mg/kg of *A. millefolium* extract over 22 days demonstrated transient antifertility effects. Dispersed immature cells on the basal membrane, along with reduced cellular vacuolization and accumulation within the seminiferous tubules, were detected at 400 mg/kg dose levels following intraperitoneal administration. At an 800 mg/kg dose level, administered intraperitoneally, a markedly decreased sperm cell concentration was induced, along with thickening of the seminiferous tubules’ basal membrane, reduced cellular accumulation within the seminiferous tubules, significant disorganization, and the presence of degenerating cells. At an orally dosed 800 mg/kg/day, thickening of the basal membrane, as well as cellular disarray, was seen (28).

Similarly, *Achillea santolina*, when administered as a hydroalcoholic extract by intraperitoneal injection at 300 mg/kg over 20 days, exhibited anti-spermatogenic activity parallel to that of *A. millefolium* in mice (29). The detected modifications included germinal epithelium disordering, exfoliation of immature germ cells, necrotic germ cells, and amplified metaphases of the germinal epithelium in the seminiferous tubules.

It was claimed that *A. tenuifolia* (100, 150, and 200 mg/kg) on anxiety-related behaviors and reproductive health in male rats under chronic restraint stress for 21 days was examined. The extract improved sperm quality and fertility by increasing total count, motility, and viability. Additionally, it had a favorable effect on the seminal vesicles, testicular, and final body weights. *A. tenuifolia* also decreased MDA levels, which is consistent with increased antioxidant ability (30).

From the previous findings, *Achillea* species demonstrate both beneficial and detrimental effects on male fertility, depending upon the dosage and particular species involved. *A. millefolium* demonstrates potential in safeguarding against testicular toxicity; nonetheless, elevated doses may result in pronounced antispermatogenic effects. *A. santolina* likewise exhibits antispermatogenic effects (31). These findings underscore the significance of dosage and species-specific reactions in the therapeutic use of *Achillea* extracts. To comprehensively elucidate the mechanisms of action and enhance the medicinal application of these plants, further research investigations are still needed.

Depending upon the previous research findings that highlight the necessity for further investigation into the effects of *A. fragrantissima* beyond its traditional medicinal uses, we now hypothesize that AFG extract could modulate male reproductive performance and influence the immune-related responses. The current study seeks to investigate the outcomes of *A. fragrantissima* on male fertility and immune function, consequently augmenting our comprehension of *A. fragrantissima* as a prospective therapeutic agent.

## 2 Materials and methods

### 2.1 *Achillea Fragrantissima* aerial parts collection, authentication, and extraction

*Achillea fragrantissima* plant was obtained from a local herbal market in Arish, Northern Sinai, Egypt. Dr. Marwa M. El Demerdash, from the Botany and Microbiology Department, Faculty of Science, Zagazig University, carried out the botanical identification using taxonomic and morphological details. The sample with voucher number (Mans-0010106007) was deposited in the Herbarium of the Botany Department, Faculty of Science, Mansoura University, Egypt. The aerial parts of AFG were thoroughly milled using a high-speed milling machine to crush the dried material finely. Approximately 200 g of finely pulverized powder was then extracted in a Soxhlet apparatus using 1 liter of 99% pure ethanol for 6 h, in accordance with the method described by Adebiyi et al. (32). A semi-solid, viscous material (brownish gum) was created by concentrating the *A. fragrantissima* extract at 40 °C under reduced pressure using a rotary evaporator. The resulting AFG extract had a residual yield of 17.6 g per 100 g of dried powder. The resultant extract was stored in sterile, opaque vials at 4 °C until needed, after being reconstituted with propylene glycol in a brown bottle.

HPLC previously analyzed the AFG ethanolic extract used in the present trial. The primary detected phenolic acids were ferulic acid (2882.06 μg/g), vanillin (1189.76 μg/g), chlorogenic acid (10561.30 μg/g), catechin (16,872 μg/g), syringic acid (6922.31 μg/g), pyro catechol (690.81 μg/g), caffeic acid (931.09 μg/g), gallic acid (452.78 μg/g), methyl gallate (469.71 μg/g), ellagic acid (368.55 μg/g), and cinnamic acid (106.92 μg/g). The notable flavonoids identified by HPLC were mainly naringenin (22243.28 μg/g), diadzein (7523.45 μg/g), quercetin (4375.62 μg/g), rutin (3084.89 μg/g), kaempferol (1127.68 μg/g), hesperetin (729.72 μg/g), and apigenin (219.86 μg/g).

### 2.2 Ethical approval

All procedures involving the usage and maintenance of lab animals for research purposes in the study protocol reviewing and approval by ZU-IACUC committee in accordance with the United Kingdom. Animals [Scientific Procedures] Act, 1986 and related guidelines, EU Directive 2010/63/EU for animal experiments, the National Research Council’s Guide for the Care and Use of Laboratory Animals [NIH Publications No. 8023, revised 1978] and in compliance with the ARRIVE guidelines with approval number: ZU-IACUC/2/F/108/2025.

### 2.3 Management of laboratory animals and study protocol

Herein, we used 45 male Sprague–Dawley rats, which were procured from the experimental animal components of the Faculty of Veterinary Medicine at Zagazig University. Around 230 ± 20 g was the average weight of rats. This trial was conducted in the Department of Forensic Medicine and Toxicology at Zagazig University’s Faculty of Veterinary Medicine. The rats spent 14 days acclimating to the lab setting. Animals were maintained in plastic cages with unlimited access to water and feed. Conditions in the lab included a photoperiod of 12 h of light and 12 h of dark, an average relative humidity of 50 to 60%, and an average temperature of 22 °C to 28 °C.

Following weighing, the rats were assigned at random into three sets, with 15 rats in each set. The 1st control set received physiological saline. The 2nd (AFG-L) and 3rd (AFG-H) sets were administered the low dose (500 mg/kg B.wt.) and high dose (1,000 mg/kg B.wt.), respectively, of AFG extract. Rats were gavaged via a gastric tube daily for 4 weeks. Throughout the experiment, the rats were carefully observed for any unusual behavior, signals of discomfort, or mortalities. Dose was selected according to Alhomaid et al. (17), who reported that *A. fragrantissima* extract at 500 mg/kg significantly enhanced immune-related responses. El-Ashmawy et al. (33) reported that oral administration of methanol extract of *A. fragrantissima* at 1000 mg/kg body weight produced moderate efficacy (40%) against *Trypanosoma evansi* infection in rats, besides it reversed reductions in packed cell volume, hemoglobin, and total leukocyte count, and normalized OS markers in infected rats.

The selection of doses was based upon previous studies involving *A. fragrantissima* and related *Achillea* species that utilized different dosages to assess biological effects. The oral LD50 values for *A. fragrantissima* extract were documented to exceed 2000 mg/kg. A rat study showed no acute toxicity at oral dosages of 2000–5,000 mg/kg and indicated that *A. fragrantissima* has protective effects against ethanol-induced stomach damage, presumably due to its antioxidant properties (34), or at doses above 4 g/kg (35). A rat cardiotoxicity model employed dosages of 400 mg/kg and 800 mg/kg of *A. Fragrantissima* pretreatment mitigates adriamycin-induced heart damage; both dosages produced beneficial antioxidant and anti-inflammatory effects, with the higher dose demonstrating superior benefits (19). In rat experiments, the oral administration of *A. fragrantissima* extract at dosages of 300 and 500 mg/kg/day showed significant immunomodulatory effects, with larger doses preferred for the restoration of immunological responses *in vivo* (17, 36). The selection of 500 mg/kg as a lower test dose enables the assessment of dose–response relationships and the identification of threshold effects, while 1,000 mg/kg serves as the regulatory limit, maximizing the likelihood of detecting adverse reproductive outcomes without exceeding safety thresholds (17, 37). Ethanolic and aqueous extracts of *A. fragrantissima*, when repeatedly administered over an extended period, exhibited strong *in vitro* antimalarial activity with no observed adverse effects or toxicity in rats. These extracts are described as well-tolerated substances with a large margin of safety (38).

### 2.4 Behavioral assessments

#### 2.4.1 Tube dominance investigation

The Lindzey test is a behavioral assessment designed to evaluate aggression in rats by placing two subjects in a narrow, clear, plastic tube that is too small for them to pass each other. Each rat enters from opposite ends, and the more aggressive rat must push the other backwards out of the tube. The test, typically conducted with a 30 cm long tube of an appropriate diameter, measures the number of successful dominations by each rat over multiple trials. Before testing, the rodents are habituated to the apparatus and trained to traverse the tube individually, often with the incentive of a food reward. During the actual test, once both rats are at either end of the tube, the one that forces the other out is considered dominant, while the one that is pushed out is deemed subordinate (39).

#### 2.4.2 Hole-board investigation

A wooden cube board measuring 50 × 50 cm was utilized, featuring four holes evenly distributed in each quarter. Modifications were made to assess exploratory behavior via determining how long time rats took for the head to enter the holes and the frequency of head entrances over a three-minute observation period (40).

#### 2.4.3 Forced swimming and swimmer performance investigation

A procedure was used on rats to evaluate their attention, awareness, and good behavior reasonably. All rats from every experimental set were enforced to swim in a cylindrical tank made of glass, 50 cm in tall, 25 cm in width, full with 35 cm of water at 25 °C, rats hold to swim over 3 mins (41), then duration in seconds was recorded overall actual swimming, scoring of swimming performance via the variable position of their heads and noses relative to the water surface (42) with 0 representing head and nose are submerged, 1 representing the nose directly above the water surface, 2 representing head and nose are above the water surface but ear is below, 3 representing mid ear below water surface, and 4 representing the mid ear directly above water surface.

#### 2.4.4 Standard opponent investigation

This test is used to evaluate sexual behavior. Two randomly selected rats, who had not met previously, were put in a cage, and the behaviors they exhibited over a period of time were recorded. Each rat’s sexual behavior, like sniffing, jumping, etc., was documented and employed to evaluate the rat’s total social dominance (43).

#### 2.4.5 Modified Y-maze test

This test was utilized to assess short-term memory in this study. This Y-shaped maze comprises three arms and a core zone. We designated these arms as A, B, and C, each measuring 35 cm in length and 10 cm in width, and positioned the maze 25 cm above the ground. All rats were oriented toward arm A in the middle region, ensuring uniformity in their positioning. The duration of the session was 5 mins for a single rat. The arms into which the rats were placed were meticulously observed. The absence of all four paws of a mouse within the arm was not considered an entry. Following a 5-min interval, the rats were removed from the maze, which was subsequently sanitized with 10% ethanol in preparation for the following subject. When the rat sequentially moved all three arms (ABC, ACB, CAB, etc.), it was deemed a correct alternation. The percentage of spontaneous alternations (SAP) was determined by means of:

Total arm entries (TAE) and Spontaneous alternation percentage (SAP) where.
SAP = [(Number of alternations) / (TAE-2)] ∗100 (44).

### 2.5 Sampling

After the 30-day experimental study, the rats’ body weights were recorded, and they had fasted overnight. Six rats were chosen randomly from each group, sterile blood samples were drawn from each group from the retro-orbital venous plexus using EDTA tubes for hematological evaluation, heparinized tubes for the phagocytosis assay, and anticoagulant-free tubes for serum separation, kept to coagulate, centrifuged for 15 mins at 3599 × g rpm to separate sera for subsequent biochemical measurements. The sera samples were preserved at ^−^20 °C. Rats were then euthanasized by decapitation after pentobarbital sodium (100 mg/kg B.wt.) was intraperitoneally injected to induce anesthesia. After the decapitation of each rat, testes and spleen specimens were dissected and directly preserved in TRIzol at −80 °C for the RT-PCR molecular analysis. Testes and spleen specimens were collected, cleaned with physiological saline, and homogenized by tissue homogenizer (Potter-Elvehjem, Thomas Scientific, Swedesboro, NJ, United States). About 0.5 g of specimen was homogenized in 4.5 mL of cold potassium chloride and centrifuged at 4 °C for 15 mins at 664 × g. The obtained supernatant solutions were used for the estimation of OS biomarkers. Finally, the additional testes and spleen specimens were preserved for histopathological examination in neutral buffered formalin (10%) at room temperature.

### 2.6 Semen valuation

Immediately, the testes were excised, devoid of any connective or adipose tissues, and subsequently weighed; the relative testicular weight was computed utilizing the following formula:

Relative testis weight = [testis weight/body weight] * 100 (45).

The cauda epididymis was excised post-euthanasia and treated with preheated 2 mL physiological saline at 37 °C, which was added (46) for analysis of sperm quality parameters. Sperm count, motility %, malformations %, and viability % were assessed. The motile sperm percentage was calculated by an independent scoring scheme with a range of 0 to 100%. Motility was categorized into two types: motile (with rapid or slow linear or circular movements) and immotile (without any movement). To determine sperm count, the epididymal suspension was diluted with five milliliters of physiological saline and was gently shaken for 5 mins at 37 °C to allow sperm to diffuse into the diluent. Sperms were counted by a Neubauer hemocytometer (Deep 0.1 mm, LABART, Germany) at 40 × magnification in an ordinary microscope. The mean was then multiplied by 10^6^ to determine the total count of sperm cells per mL of semen. The sperm were totaled in their entirety, including the heads plus tails. Aberrant sperm percentage, the sperm were stained for 5 to 10 min with eosin/nigrosin and allowed to dry. In each sample, 2 to 3 hundred sperm were numbered for each rat, and the proportion of aberrant sperm was calculated. For the determination of sperm viability, sperm eosin/nigrosin staining was utilized to evaluate the sperm’s vitality. The production of nigrosin and eosin (Merck, Darmstadt, Germany) involved refined water. One volume of sperm suspension was mixed with two volumes of 1% eosin. An equivalent volume of nigrosin was added to this mixture after 30 s. After that, smears were created and seen by a microscope at 40 ×. In this manner, non-viable sperms are stained red, while the viable sperms are not stained.

### 2.7 Phagocytosis assay and evaluation of hematological indicators

In accordance with (47), the activity of phagocytosis. One mL of the heat-inactivated *Candida albicans (C. albicans)* was mixed with one mL of the calibrated solution of live leukocytes in a sterile plastic tube. The tubes were incubated for over 30 mins at 27 °C in a humidified CO_2_ 5% incubator. Pasteur pipettes were used to collect the supernatants after the tubes had been centrifuged for 5 mins at 2500 rpm. A small amount of the supernatants was then utilized to resuspend the sediment. The deposit was used to make slide smears, which were left to air-dry before applying Leishman’s staining.

Under a microscope, phagocytically active cells in 10 microscopic regions were randomly calculated using the oil immersion lens. Both the quantity of *C. albicans* within every phagocytic cell and the count of phagocytic cells that adhered or ingested the *C. albicans* were counted. The phagocytic activity was measured using the following formulas. The phagocytic percentage (Ph%) = (count of phagocytes engulfing *C. albicans* /total count of phagocytic cells) × 100. Phagocytic index (PhI) = (total count of engulfed *C. albicans* /count of phagocytic cells engulfing *C. albicans*) × 100 according to Muniz-Junqueira et al. (48).

A Hemascreen 18 automatic cell counter (Hospitex, Osmannoro-Sesto Fiorentino, Italy) was used to immediately inspect the collected blood samples in EDTA tubes for determination of total erythrocyte count (RBC count; 10^6^/mL), hematocrit value (HCT), hemoglobin content (Hb), mean corpuscular volume (MCV), mean corpuscular hemoglobin concentration (MCHC), and total leukocytes, granulocytes, lymphocytes, and monocytes (49).

### 2.8 Serum biochemical and oxidative stress-related indicators in spleen and testes measurements

The concentrations of total cholesterol (TC), triglycerides (TGs), high-density lipoproteins (HDL-C), and low-density lipoproteins (LDL-C) in serum were determined by colorimetric kits of SPINREACT, Girona, SPAIN with no. Ref: SP4102, Ref: MX41031, Ref: 1001095, and Ref: 41023, respectively. Also, VLDL-C was calculated as TG / 5 (HATA and NAKAJIMA 1986). A commercial rat ELISA kit from United States R&D Systems, Inc. (catalog No: R6000B), was used to determine the serum concentrations of interleukin (IL-6). At the same time, the rat ELISA kit from Cusabio Biotech United States (catalog no: CSB-E11987r-IS) was used to determine serum levels of tumor necrosis factor (TNFα). Commercial rat ELISA kits from Cusabio Biotech, United States (catalog no: CSB-E05100r, CSB-E06869r, CSB-E12654r, and CSB-E05110r) were used to determine the follicle-stimulating hormone (FSH), testosterone (TEST), estradiol (E2), and luteinizing hormone (LH) concentrations in serum as directed by the manufacturer.

For the oxidative stress indicators in spleen and testes homogenates, the Cell Biolabs’ OxiSelect™ Assay Kits were used to determine the total antioxidant capacity (TAC), Superoxide Dismutase Activity (SOD), and TBARS (MDA Quantitation) concentrations in the testicles and spleen homogenates with catalog no. (STA-360, STA-340, and STA-330). The Cell Biolabs’ OxiSelect™ ELISA Kits were used to measure the levels of protein carbonyl (PCO) and the oxidatively damaged DNA ELISA Kit (8-OHdG Quantitation) with catalog no. (STA-310 and STA-320). The Rat Lactate Dehydrogenase (LDH) ELISA Kit (Ref: MX41214) from SPINREACT, SPAIN was used to measure the amounts of LDH in the testes. Specific Rat Glutathione Peroxidase (GSH-PX1) ELISA Kit of My BioSource Co. (Cat. No. MBS701677).

### 2.9 Transcriptional investigation of testosterone synthesis pathway and immune-related genes in testes and spleen by quantitative real-time PCR

The TRIzol reagent (Invitrogen; Thermo Fisher Scientific, Waltham, MA, USA, Catalog No. 15596026) was utilized for extracting total RNA. Total RNA (1 μg) was reverse transcribed into cDNA by the HiSenScript™ RH (−) cDNA Synthesis Kit (iNtRON Biotechnology Co., South Korea). The gene expression study was conducted using 5x HOT FIRE Pol EvaGreen qPCR Mix Plus (Solis BioDyne, TARMu, Estonia) and a Mx3005P Real-Time PCR System (Agilent Stratagene, USA), according to the directions provided by the producer. The PCR cycling process began with a 15-min denaturation step at 95 °C and continued with 40 cycles of denaturation for 30 s, annealing for 60 °C for 60 s, and extension for 72 °C. Sangon Biotech (Beijing, China) manufactured the oligonucleotide-specific primer sequences for the *StAR, CYP11A1*, *CYP17A1*, *CYP19A1*, *HSD17B3*, and *Caspase-3* genes in the testicular tissue and the *CD3*, *CD8*, *CD4*, *CD20*, *IL-10*, and *Caspase-3* in the splenic tissue. Validation of primer efficiency was carried out according to Sreedharan et al. (50), ensuring reliable amplification of the target. The sequences are shown in Table 1. We used GAPDH as a reference gene to standardize the target genes’ expression levels. The comparative 2^−ΔΔCT^ (Ct: cycle threshold) procedure was employed for the gene expression relative fold changes assessments (51).

**Table 1:** Primer sequences, accession number, and product size for the quantitative RT-PCR for the analyzed genes in the testes and spleen tissues.

Gene	Forward primer (5′–3′)	Reverse primer (5′–3′)	bp	Accession no.
*StAR*	CCCAAATGTCAAGGAAATCA	AGGCATCTCCCCAAAGTG	187	NM_031558.3
*CYP11A1*	AAGTATCCGTGATGTGGG	TCATACAGTGTCGCCTTTTCT	127	NM_017286.3
*CYP17A1*	TGGCTTTCCTGGTGCACAATC	TGAAAGTTGGTGTTCGGCTGAAG	90	NM_012753.2
*CYP19A1*	GCTGAGAGACGTGGAGACCTG	CTCTGTCACCAACAACAGTGTGG	178	NM_017085.2
*HSD17B3*	ATT ACC TCC GTA GTC AAG A	TAT TCC ACA TTC AAA GCC T	167	NM_054007.1
*Casp-3*	GGTGTCCCTAAACCTGGGTG	TCACTTTTCTTCGCCCCTCC	149	NM_001436900.1
*CD3*	AAAGGTTTGGCTGGCCTCTT	GCCATCTCCTTGGCTGTCAT	108	NM_001077646.2
*CD8*	ACTCACGGAGTGTGCTGAAG	CAGTCATGCTGCCCTACCAA	137	NM_031539.2
*CD4*	AGAAAGGACTGGCCAGAGAC	CTGAAAGAGAAGCCTCGGCA	73	NM_012705.1
*CD20*	CCAGCTGATCTCAGCAGTGAA	TTTTGAGCAGGTTGCATGGC	161	NM_001399452.1
*IL-10*	GCTCAGCACTGCTATGTTGC	TTGTCACCCCGGATGGAATG	76	NM_012854.2
*Gapdh*	ACGGGAAACCCATCACCATC	ACGACATACTCAGCACCAGC	79	NM_017008.4

StAR, steroidogenic acute regulatory protein; CYP11A1, cytochrome P450, family 11, subfamily a, polypeptide 1; CYP17A1, cytochrome P450, family 17, subfamily a, polypeptide 1; CYP19A1, cytochrome P450, family 19, subfamily a, polypeptide 1; HSD17B3, hydroxysteroid (17-beta) dehydrogenase 3; Casp-3, caspase 3; CD3, cluster of differentiation 3 gamma subunit of T-cell receptor complex; CD8, cluster of differentiation 8 subunit beta; CD4, cluster of differentiation molecule 4; CD20, cluster of differentiation 20 membrane spanning 4-domains A1; IL-10, interleukin 10; GAPDH: glyceraldehyde-3-phosphate dehydrogenase.

### 2.10 Testes and spleen histopathological investigation

The left testis and spleen of 10 rats/group were collected and well-kept in 10% neutral buffered formalin for over 48 h. Samples were then dehydrated in ethanol (70–100%), preserved with two rounds of xylene (1 h for each), equipped for paraffin impregnation and embedding, sectioned into tissue slices (five-micron), and stained with hematoxylin and eosin (52). Sections were inspected under a microscope, and tissue structure alterations were documented. For morphometric analysis and quantitative lesion evaluation of the testicular and splenic tissues, one slide per rat was chosen. A Leica® microscope with an AmScope digital camera was used to choose five testicular and splenic images/slides at random. The chosen images/slides were round or almost round in shape and did not overlap. Five images or slides were obtained at 10 ×, then five more at 40 × .

Histopathological lesions scoring was measured using a scoring method of Gibson-Corley et al. (53). Thirty tubular testicular and splenic sections per rat from all experimental sets (10 rats per set), chosen at random, were investigated by high-power fields (HPF, 40 ×). The following is how lesions were assessed: the number of lesions is indicated by a score of: 0 represents no lesion; 1 represents 1–25% impacted; 2 represents 26–50% affected; 3 represents 51–75% damaged; and 4 represents 76–100% affected tissues.

### 2.11 Data statistics approach

To assess the normality, the Kolmogorov–Smirnov test and the Shapiro–Wilk test were used, revealing no significant deviations from normality. Homogeneity of variance was assessed using Levene’s test indicated a substantial violation of equal variances. Consequently, we employed Welch’s one-way ANOVA, which does not presume homogeneity of variance, using the IBM SPSS Statistics, version 22 (IBM; Armonk, New York, USA). Following Welch, ANOVA Post-hoc comparisons were conducted using the Games–Howell test. Data are presented as mean ± standard error mean (SEM). A significance threshold of *p < 0.05* was applied. Graph Pad Prism version 10 (Graph Pad Software, San Diego, CA, USA) was used to visualize the data. The Kruskal−Wallis test was employed to examine the histopathological results, followed by Dunn’s multiple comparisons test.

## 3 Results

### 3.1 Effects of *Achillea Fragrantissima* extract administration on behavioral observations

. The outcomes of the AFG on behavioral assessments are shown in Table 2. The time of feeding differed significantly across groups, W (2, 9.87) = 33.51, *p* < 0.001. The AFG-L (107.67 ± 2.64; 34.33, 95% CI [22.91, 45.75], *p* < 0.001) and AFG-H (99.00 ± 2.39; 25.67, 95% CI [14.59, 36.74], *p* < 0.001) had significantly increased feeding time than the C group (73.33 ± 3.19), which suggests improved appetite. There was no significant difference between AFG-L and AFG-H groups (8.67, 95% CI [1.12–18.46], *p* = 0.083). The feeding frequency differed significantly between groups, W (2, 7.35) = 9.42, *p* = 0.009. The AFG-L (6.33 ± 1.11; 4.67, 95% CI [1.06, 8.27], *p* = 0.018) and the AFG-H (3.33 ± 0.76; 4.67, 95% CI [1.06, 8.27], *p* = 0.018) groups had significantly higher feeding frequency than the C group (1.67 ± 0.21) suggests neutral effect of AFG extract administration. There was no significant difference between AFG-L and AFG-H groups (3.00, 95% CI [−0.78, 6.78], *p* = 0.121).

**Table 2:** Effect of *A. fragrantissima* extract oral administration on behavioral observations of adult male Sprague Dawley rats for 30 days.

Behavior test	Response measure	W (df1, df2)	*p*-value	C	AFG-L	AFG-H	*Post-hoc* Games Howell (Significant pairs)
Feeding	T	33.51 (2, 9.87)	0.000	73.33 ± 3.19 ^b^	107.6 ± 2.64 ^a^	99.00 ± 2.39 ^a^	2 > 1 (34.33, 95% CI [22.91, 45.75], *p* < 0.001); 3 > 1 (25.67, 95% CI [14.59, 36.74], *p* < 0.001); 2 > 3 (8.67, 95% CI [1.12–18.46], *p* = 0.083)
	F	9.42 (2, 7.35)	0.009^**^	1.67 ± 0.21 ^b^	6.33 ± 1.11^a^	3.33 ± 0.76 ^ab^	2 > 1 (4.67, 95% CI [1.06, 8.27], *p* = 0.018); 3 > 1 (1.67, 95% CI [−0.78, 4.12], *p* = 0.170); 2 > 3 (3.00, 95% CI [−0.78, 6.78], *p* = 0.121)
Drinking	T	4.87 (2, 8.77)	0.038	16.00 ± 1.83 ^b^	27.67 ± 3.11 ^a^	19.00 ± 1.09 ^ab^	2 > 1 (11.67, 95% CI [1.40, 21.94], *p* = 0.028); 3 > 1 (3.00, 95% CI [3.05, 9.05], *p* = 0.380); 2 > 3 (8.67, 95% CI [1.33, 18.67], *p* = 0.084).
	F	8.35 (2, 9.61)	0.008	3.00 ± 0.37 ^a^	1.33 ± 0.21^b^	1.33 ± 0.21^b^	1 > 2 (1.67, 95% CI [0.46, 2.87], *p* = 0.010); 1 > 3 (1.67, 95% CI [0.46, 2.87], *p* = 0.010); 2 = 3 (0.00, 95% CI [0.82, 0.82], *p* = 1.00)
Hole-board	T	5.21 (2, 8.52)	0.033	10.33 ± 1.12 ^b^	14.67 ± 2.43 ^ab^	19.67 ± 2.74 ^a^	2 > 1 (4.33, 95% CI [3.54, 12.21], *p* = 0.299); 3 > 1 (9.33, 95% CI [0.49, 18.17], *p* = 0.040); 3 > 2 (3.67, 95% CI [5.07, 15.07], *p* = 0.395).
	F	3.10 (2, 9.03)	0.094	2.67 ± 0.55 ^a^	1.33 ± 0.21 ^a^	1.16 ± 0.17 ^a^	1 > 2 (1.33, 95% CI [−0.46, 3.13], *p* = 0.138); 1 > 3 (1.50, 95% CI [−0.29, 3.29], *p* = 0.094); 2 > 3 (0.67, 95% CI [−0.58, 0.91], *p* = 0.813)
Swimming performance test	T	9.92 (2, 9.93)	0.004	6.00 ± 0.63 ^b^	9.00 ± 0.73 ^a^	10.33 ± 0.76 ^a^	2 > 1 (3.00, 95% CI [0.34, 5.66], *p* = 0.028); 3 > 1 (4.33, 95% CI [1.61, 7.06], *p* = 0.004); 3 > 2 (1.33, 95% CI [−1.56, 4.22], *p* = 0.445)
	P	1.04 (2, 9.08)	0.391	4.00 ± 0.00 ^a^	4.01 ± 0.00 ^a^	4.01 ± 0.00 ^a^	2 > 1 (0.003, 95% CI [−0.009, 0.015], *p* = 0.719); 3 > 1 (0.007, 95% CI [−0.007, 0.020], *p* = 0.383); 2 > 3 (0.003, 95% CI [−0.012, 0.019], *p* = 0.833)
Tube dominance	FW T	20.06 (2, 9.27)	0.000	47.83 ± 7.60 ^b^	107.00 ± 4.91^a^	84.33 ± 9.44 ^a^	2 > 1 (59.17, 95% CI [33.66, 84.68], *p* < 0.001); 3 > 1 (36.50, 95% CI [3.01, 69.99], *p* = 0.034); 2 > 3 (22.67, 95% CI [−8.17, 53.50], *p* = 0.149)
	FW F	1.34 (2, 8.56)	0.312	2.33 ± 0.42 ^a^	3.33 ± 0.55 ^a^	2.33 ± 0.21 ^a^	2 > 1 (1.00, 95% CI [−0.94, 2.94], *p* = 0.366); 3 = 1 (0.00, 95% CI [−1.37, 1.37], *p* = 1.00); 2 > 3 (1.00, 95% CI [−0.79, 2.79], *p* = 0.284)
	BW T	11.94 (2, 9.67)	0.002	100.33 ± 7.69 ^a^	45.00 ± 8.73 ^b^	90.00 ± 5.73 ^a^	1 > 2 (55.33, 95% CI [23.36, 87.31], *p* < 0.002); 1 > 3 (10.33, 95% CI [16.34, 37.01], *p* = 0.551); 2 > 3 (45.00, 95% CI [15.62, 74.38], *p* < 0.005)
	BW F	1.42 (2, 8.77)	0.291	2.67 ± 0.55 ^a^	1.66 ± 0.21 ^a^	2.00 ± 0.36 ^a^	1 > 2 (1.00, 95% CI [−0.79, 2.97], *p* = 0.284); 1 > 3 (0.67, 95% CI [−1.21, 2.54], *p* = 0.596); 3 > 2 (0.33, 95% CI [−0.87, 1.53], *p* = 0.719)
Standard opponent test	Enter speed T	58.36 (2, 7.27)	0.000	56.67 ± 3.74 ^a^	13.33 ± 0.91 ^b^	21.33 ± 4.77 ^b^	1 > 2 (43.33, 95% CI [31.24, 55.42], *p* < 0.000); 1 > 3 (35.33, 95% CI [18.56, 52.11], *p* < 0.001); 3 > 2 (8.00, 95% CI [−7.41, 23.41], *p* = 0.306)
	Enter speed F	4.77 (2, 6.67)	0.052	1.34 ± 0.21 ^a^	2.01 ± 0.003 ^a^	1.67 ± 0.42 ^a^	2 > 1 (0.67, 95% CI [−0.025, 1.36], *p* = 0.057); 3 > 1 (0.33, 95% CI [−1.04, 1.69], *p* = 0.770); 2 > 3 (0.34, 95% CI [−1.03, 1.71], *p* = 0.716)
	Body sniff T	15.82 (2, 9.08)	0.001	16.67 ± 1.83 ^b^	33.83 ± 2.35 ^a^	21.33 ± 1.12 ^b^	2 > 1 (17.17, 95% CI [8.89, 25.44], *p* < 0.001); 3 > 1 (4.67, 95% CI [1.44, 10.77], *p* = 0.135); 2 > 3 (12.50, 95% CI [4.85, 20.15], *p* < 0.005)
	Body sniff F	7.49 (2, 8.78)	0.013	1.33 ± 0.21 ^b^	3.67 ± 0.56 ^a^	2.00 ± 0.36 ^ab^	2 > 1 (2.33, 95% CI [0.54, 4.13], *p* < 0.016); 3 > 1 (0.67, 95% CI [−0.538, 1.87], *p* = 0.307); 2 > 3 (1.67, 95% CI [−0.209, 3.54], *p* = 0.081)
	Jump T	29.26 (2, 6.84)	0.000	46.00 ± 2.03 ^a^	38.33 ± 1.66 ^b^	31.67 ± 0.21 ^c^	1 > 2 (7.67, 95% CI [0.41, 14.92], *p* = 0.039); 1 > 3 (14.33, 95% CI [7.73, 20.93], *p* = 0.002); 2 > 3 (6.67, 95% CI 1[1.26, 12.07], *p* = 0.023)
	Jump F	9.43 (2, 9.86)	0.005	1.16 ± 0.16 ^b^	2.33 ± 0.21 ^a^	1.33 ± 0.21 ^b^	2 > 1 (1.17, 95% CI [0.42, 1.91], *p* < 0.004); 3 > 1 (0.17, 95% CI [0.58, −0.91], *p* = 0.813); 2 > 3 (1.00, 95% CI [0.18, 1.82], *p* < 0.018)
	Anal sniff T	23.07 (2, 9.60)	0.000	13.33 ± 1.47 ^b^	25.67 ± 1.12 ^a^	16.67 ± 1.83 ^b^	2 > 1 (12.33, 95% CI [7.20, 17.47], *p* < 0.000); 3 > 1 (3.33, 95% CI [−3.18, 9.84], *p* = 0.372); 2 > 3 (9.00, 95% CI [2.89, 15.11], *p* = 0.007)
	Anal sniff F	1.34 (2, 9.61)	0.308	1.33 ± 0.21 ^a^	2.00 ± 0.36 ^a^	1.67 ± 0.21 ^a^	2 > 1 (0.67, 95% CI [−0.54, 1.87], *p* = 0.307); 3 > 1 (0.33, 95% CI [−0.48, 1.15], *p* = 0.525); 2 > 3 (0.33, 95% CI [−0.87, 1.54], *p* = 0.719)
Exploratory T		27.07 (2, 8.97)	0.000	43.67 ± 3.90 ^a^	16.33 ± 1.12 ^b^	13.33 ± 0.92 ^b^	1 > 2 (27.33, 95% CI [14.76, 39.91], *p* < 0.001); 1 > 3 (30.33, 95% CI [17.73, 42.93], *p* < 0.001); 2 > 3 (3.00, 95% CI [−0.98, 6.99], *p* = 0.147)
Grooming F		18.94 (2, 9.47)	0.000	3.67 ± 0.42 ^a^	1.33 ± 0.21^b^	0.66 ± 0.21 ^b^	1 > 2 (2.33, 95% CI [0.96, 3.71], *p* < 0.004); 1 > 3 (3.00, 95% CI [1.63, 4.37], *p* < 0.001); 2 > 3 (0.67, 95% CI [−0.15, 1.48], *p* = 0.113)
Aggression F		24.22 (2, 10.00)	0.000	2.33 ± 0.21^a^	0.67 ± 0.21 ^b^	0.33 ± 0.21 ^b^	1 > 2 (1.67, 95% CI [0.85, 2.48], *p* < 0.001); 1 > 3 (2.00, 95% CI [1.18, 2.82], *p* < 0.000); 2 > 3 (0.33, 95% CI [−0.48, 1.15], *p* < 0.525)
Wall peck T		39.79 (2, 7.87)	0.000	44.33 ± 4.40 ^a^	11.33 ± 1.64 ^b^	5.00 ± 0.73 ^c^	1 > 2 (33.00, 95% CI [18.82, 47.18], *p* < 0.001); 1 > 3 (39.33, 95% CI [25.08, 53.59], *p* < 0.001); 2 > 3 (6.33, 95% CI [1.01, 11.66], *p* < 0.024)
Modified maize test (SAP %)		6.93 (2, 9.89)	0.013	16.66 ± 10.54 ^b^	71.67 ± 11.00 ^a^	63.89 ± 13.72^ab^	2 > 1 (55.00, 95% CI [13.21, 96.79], *p* = 0.012); 3 > 1 (47.22, 95% CI [−0.73, 95.17], *p* = 0.053); 2 > 3 (7.78, 95% CI [−40.82, 56.37], *p* = 0.899)

Values are mean ± SEM of 6 animals per experimental group. Welch One-Way Analysis of Variance (ANOVA) followed by the Games-Howell test. Groups carrying the same superscript letters indicate no significant differences at the *p* < 0.05 level. T, time; F, frequency; P, performance.

The drinking time differed significantly between groups, W (2, 8.77) = 4.84, *p* = 0.038. The AFG-L (27.67 ± 3.11) had significantly longer drinking time than the C group (16.00 ± 1.83; 11.67, 95% CI [1.40, 21.94], *p* = 0.028), while in the AFG-H group (19.00 ± 1.09), drinking time did not differ significantly than the C group (3.00, 95% CI [−3.05, 9.05], *p* = 0.380), which is considered a neutral or non-adverse effect of AFG extract. There was no significant difference between AFG-L and AFG-H groups (8.67, 95% CI [1.33, 18.67], *p* = 0.084). For drinking frequency, differed significantly between groups, W (2, 9.61) = 8.35, *p* = 0.008. The AFG-L (1.33 ± 0.21) and AFG-H (1.33 ± 0.21) had a significantly decreased drinking frequency (1.67, 95% CI [0.46, 2.87], *p* = 0.010) than the C group (3.00 ± 0.37), which suggests calming and no distress. No significant difference between the AFG-L and AFG-H groups (0.00, 95% CI [0.82, 0.82], *p* = 1.00) was observed. The statistically significant enhancement of feeding duration and frequency, in addition to drinking frequency, is indicative of the beneficial effects. Findings of the hole-board test showed a significant group effect on hole-board time among groups, W (2, 8.52) = 5.21, *p* = 0.033. The AFG-H showed (19.67 ± 2.74) significantly increased hole-board time than the C group (10.33 ± 1.12), (9.33, 95% CI [0.49, 18.17], *p* = 0.040). No significant differences were observed between the AFG-L (14.67 ± 2.43) and the C group (4.33, 95% CI [3.54, 12.21], *p* = 0.299) or between the AFG-L and AFG-H groups (3.67, 95% CI [5.07, 15.07], *p* = 0.395). The hole-board frequency did not differ significantly among groups, W (2, 9.03) = 3.10, *p* = 0.094. No significant differences between AFG-L (1.33 ± 0.21) and the C group (2.67 ± 0.55; 1.33, 95% CI [−0.46, 3.13], *p* = 0.138), between the AFG-H (1.16 ± 0.17) and the C group (1.50, 95% CI [−0.29, 3.29], *p* = 0.094); or between the AFG-L and AFG-H groups (0.67, 95% CI [−0.58, 0.91], *p* = 0.813) were observed that is considered a non-adverse effect of AFG extract. The enhancement of hole-board exploration duration in the AFG-H group and reduced hole-board frequencies in both the AFG-L and AFG-H groups are indicative of extract beneficial effects on exploratory behavior, suggesting an anxiolytic or exploratory-promoting effect.

The swimming time differed significantly between groups W (2, 9.93) = 9.92, *p* = 0.004. The AFG-L (9.00 ± 0.73; 3.00, 95% CI [0.34, 5.66], *p* = 0.028) and AFG-H (10.33 ± 0.76; 4.33, 95% CI [1.61, 7.06], *p* = 0.004) groups showed a significantly increased swimming time than the C group (6.00 ± 0.63). No significant difference between the AFG-L and AFG-H groups (1.33, 95% CI [−1.56, 4.22], *p* = 0.445). Both AFG-L and AFG-H significantly enhanced swimming duration that is indicative of the beneficial effects. No significant difference in swimming performance among the groups, W (2, 9.08) = 1.04, *p* = 0.391. Differences between the AFG-L and the C groups (0.003, 95% CI [−0.009, 0.015], *p* = 0.719), between the AFG-H and the C groups (0.007, 95% CI [−0.007, 0.020], *p* = 0.383), and between the AFG-L and AFG-H groups (0.003, 95% CI [−0.012, 0.019], *p* = 0.833) were not significant. Swimming time increased without improvement of swimming performance, considered as neutral behavior, not beneficial or harmful.

Outcomes of the tube dominance test revealed a significant difference in forward walking time between the groups, W (2, 9.27) = 20.06, *p* < 0.001. The AFG-L group (107.00 ± 4.91; 59.17, 95% CI [33.66, 84.68], *p* < 0.001) and the AFG-H group (84.33 ± 9.44; 36.50, 95% CI [3.01, 69.99], *p* = 0.034) showed significantly elongated forward walking time than the C rats (47.83 ± 7.60). There was no significant difference between the AFG-L and AFG-H (22.67, 95% CI [−8.17, 53.50], *p* = 0.149) groups. The frequencies of forward walking did not differ significantly among groups, W (2, 8.56) = 1.34, *p* = 0.312. No significant difference between the AFG-L (3.33 ± 0.55) and the C groups (2.33 ± 0.42; 1.00, 95% CI [−0.94, 2.94], *p* = 0.366), between the AFG-H (2.33 ± 0.21) and the C groups (2.33 ± 0.42; 0.00, 95% CI [−1.37, 1.37], *p* = 1.00), or between the AFG-L and AFG-H groups (1.00, 95% CI [−0.79, 2.79], *p* = 0.284).

The backward walking time differed significantly between groups, W (2, 9.67) = 11.94, *p* = 0.002. The AFG-L group (45.00 ± 8.73) showed significantly decreased backward walking time than the C rats (100.33 ± 7.69; 55.33, 95% CI [23.36, 87.31], *p* < 0.002). Meanwhile, the decrease in backward time between the AFG-H (90.00 ± 5.73) and the C group (100.33 ± 7.69; 10.33, 95% CI [16.34, 37.01], *p* = 0.551) was not significant. The AFG-L differed significantly from the AFG-H group (45.00, 95% CI [15.62, 74.38], *p* < 0.005). The frequencies of backward walking did not differ significantly across groups, W (2, 8.77) = 1.42, *p* = 0.291. No significant difference between the AFG-L group (1.66 ± 0.21) and the C group (2.67 ± 0.55; 1.00, 95% CI [−0.79, 2.97], *p* = 0.284), between the AFG-H group (2.00 ± 0.36) and the C group (2.67 ± 0.55; 0.67, 95% CI [−1.21, 2.54], *p* = 0.596), or between the AFG-L and AFG-H groups (0.33, 95% CI [−0.87, 1.53], *p* = 0.719).

When comparing the two treatment groups, AFG-L and AFG-H increased the forward walking time but decreased the backward walking time significantly (*p* < 0.05), while forward and backward walking frequencies did not differ significantly, indicating the beneficial effects of AFG extract on improving social dominance-related motor control.

Findings of the standard opponent test showed a significant difference in enter-speed time between the experimental groups, W (2, 7.27) = 58.36, *p* < 0.001. The AFG-L group (13.33 ± 0.91; 43.33, 95% CI [31.24, 55.42], *p* < 0.000) and the AFG-H group (21.33 ± 4.77; 35.33, 95% CI [18.56, 52.11], *p* < 0.001) showed significantly increased enter-speed time than the C rats (56.67 ± 3.74). There was no significant difference between the AFG-L and AFG-H (8.00, 95% CI [−7.41, 23.41], *p* = 0.306) groups. The differences between the experimental groups in enter-speed frequencies were not significant, W (2, 6.67) = 4.77, *p* = 0.052. No significant difference between the AFG-L (2.01 ± 0.003) and the C groups (1.34 ± 0.21; 0.67, 95% CI [−0.025, 1.36], *p* = 0.057), between the AFG-H (1.67 ± 0.42) and the C groups (1.34 ± 0.21; 0.33, 95% CI [−1.04, 1.69], *p* = 0.770), or between the AFG-L and AFG-H groups (0.34, 95% CI [−1.03, 1.71], *p* = 0.716).

The body sniff time differed significantly between groups, W (2, 9.08) = 15.82, *p* < 0.001. The AFG-L group (33.83 ± 2.35; 17.17, 95% CI [8.89, 25.44], *p* < 0.001) showed significantly increased body sniff time than the C rats (16.67 ± 1.83). Meanwhile, the increase in the AFG-H (21.33 ± 1.12; 4.67, 95% CI [1.44, 10.77], *p* = 0.135) was not significant than the C group. There were significant differences between the AFG-L and the AFG-H groups (12.50, 95% CI [4.85, 20.15], *p* < 0.005). The differences between the experimental groups in body sniff frequencies were significant, W (2, 8.78) = 7.49, *p* = 0.013. The AFG-L (3.67 ± 0.56) showed significantly increased body sniff frequency than the C group (1.33 ± 0.21; 2.33, 95% CI [0.54, 4.13], *p* < 0.016). There were no significant differences between the AFG-H (2.00 ± 0.36) and the C groups (1.33 ± 0.21; 0.67, 95% CI [−0.538, 1.87], *p* = 0.307), or between the AFG-L and AFG-H groups (1.67, 95% CI [−0.209, 3.54], *p* = 0.081).

The jump time was significantly different between the experimental groups, W (2, 6.84) = 29.26, *p* < 0.001. The AFG-L group (38.33 ± 1.66; 7.67, 95% CI [0.41, 14.92], *p* = 0.039) and the AFG-H group (31.67 ± 0.21; 14.33, 95% CI [7.73, 20.93], *p* = 0.002) showed significantly reduced jump times than the C rats (46.00 ± 2.03). There was significant difference between the AFG-L and AFG-H; 1 > 3; (6.67, 95% CI 1[1.26, 12.07], *p* = 0.023). The jump frequencies differed significantly between the experimental groups, W (2, 9.86) = 9.43, *p* = 0.005. The AFG-L group (2.33 ± 0.21; 1.17, 95% CI [0.42, 1.91], *p* < 0.004) showed significantly increased jump frequencies than the C rats (1.16 ± 0.16). Meanwhile, the increase in the AFG-H (1.33 ± 0.21; 0.17, 95% CI [0.58, −0.91], *p* = 0.813) was not significant than the C group. There were significant differences between the AFG-L and the AFG-H groups (1.00, 95% CI [0.18, 1.82], *p* < 0.018).

The anal sniff time differed significantly between the experimental groups, W (2, 9.60) = 23.07, *p* <0.001. The AFG-L group (25.67 ± 1.12; 12.33, 95% CI [7.20, 17.47], *p* < 0.000) showed significantly increased anal sniff time than the C rats (13.33 ± 1.47). Meanwhile, the increase in the AFG-H (16.67 ± 1.83; 3.33, 95% CI [−3.18, 9.84], *p* = 0.372) was not significant than the C group. There were significant differences between the AFG-L and the AFG-H groups (9.00, 95% CI [2.89, 15.11], *p* = 0.007). The differences between the experimental groups in anal sniff frequencies were not significant, W (2, 9.61) = 1.34, *p* = 0.308. No significant difference between the AFG-L (2.00 ± 0.36) and the C groups (1.33 ± 0.21; 0.67, 95% CI [−0.54, 1.87], *p* = 0.307), between the AFG-H (1.67 ± 0.21) and the C groups (1.33 ± 0.21; 0.33, 95% CI [−0.48, 1.15], *p* = 0.525), or between the AFG-L and AFG-H groups (0.33, 95% CI [−0.87, 1.54], *p* = 0.719).

AFG-L administration markedly increased (duration and frequency of body sniffing and anal sniffing), indicating enhanced social investigation, while reduced entry speed and jump time, signifying reduced aggression, and indicating beneficial effects of AFG extract on social interaction patterns.

The exploratory time was significantly different between the experimental groups, W (2, 8.97) = 27.07, *p* < 0.001. The AFG-L group (16.33 ± 1.12; 27.33, 95% CI [14.76, 39.91], *p* < 0.001) and the AFG-H group (13.33 ± 0.92; 30.33, 95% CI [17.73, 42.93], *p* < 0.001) showed significantly reduced exploratory times than the C rats (43.67 ± 3.90). There was no significant difference between the AFG-L and AFG-H (3.00, 95% CI [−0.98, 6.99], *p* = 0.147). The diminished exploration duration and frequency suggest lowered stress levels.

The grooming frequency was significantly different between the experimental groups, W (2, 9.47) = 18.94, *p* < 0.001. The AFG-L group (1.33 ± 0.21; 2.33, 95% CI [0.96, 3.71], *p* < 0.004) and the AFG-H group (0.66 ± 0.21; 3.00, 95% CI [1.63, 4.37], *p* < 0.001) exhibited significantly reduced grooming frequencies than the C rats (3.67 ± 0.42). There was no significant difference between the AFG-L and AFG-H (0.67, 95% CI [−0.15, 1.48], *p* = 0.113).

The aggression frequency was significantly different between the experimental groups, W (2, 10.00) = 24.22, *p* < 0.001. The AFG-L group (0.67 ± 0.21; 1.67, 95% CI [0.85, 2.48], *p* < 0.001) and the AFG-H group (0.33 ± 0.21; 2.00, 95% CI [1.18, 2.82], *p* < 0.000) exhibited significantly lower aggression frequencies than the C rats (2.33 ± 0.21). There was no significant difference between the AFG-L and AFG-H (0.33, 95% CI [−0.48, 1.15], *p* < 0.525). The diminished frequency of aggressiveness indicates a beneficial effect in social interaction.

The wall peck time was significantly different between the experimental groups, W (2, 7.87) = 39.79, *p* < 0.001. The AFG-L group (11.33 ± 1.64; 33.00, 95% CI [18.82, 47.18], *p* < 0.001) and the AFG-H group (5.00 ± 0.73; 39.33, 95% CI [25.08, 53.59], *p* < 0.001) exhibited significantly shorter wall peck time than the C rats (44.33 ± 4.40). There was no significant difference between the AFG-L and AFG-H (6.33, 95% CI [1.01, 11.66], *p* < 0.024). The diminished wall-peck time was observed in both the AFG-L and AFG-H groups, indicating reduced anxiety or stereotypic behavior.

The memory performances of the rats in the Y-maze investigation exhibited that SAP was significantly different between the experimental groups, W (2, 9.89) = 6.93, *p* = 0.013. The AFG-L group showed a significant increase in SAP % (71.67 ± 11.00) relative to that of the control rats (55.00, 95% CI [13.21, 96.79], *p* = 0.012). However, the difference between the AFG-H (63.89 ± 13.72) was not significant relative to the C group (47.22, 95% CI [−0.73, 95.17], *p* = 0.053). There was no significant difference between the AFG-L and AFG-H (7.78, 95% CI [−40.82, 56.37], *p* = 0.899). Increased SAP in the Y-maze test indicates improved working memory.

The AFG administration primarily yielded advantageous behavioral outcomes, especially at lower doses, such as improved feeding behavior, increased exploratory activity, greater social interaction, and superior memory function. Multiple measures remained constant, signifying behavioral neutrality at specific doses. A restricted set of activities exhibited dose-dependent decreases in activity-related frequencies, potentially indicating behavioral modulation instead of negative consequences. These effects are best described as dose-dependent behavioral modulation rather than negative toxicity.

### 3.2 Effects of *Achillea Fragrantissima* extract administration on final body weight, relative testes weight, and semen profile

**Figure 1:**
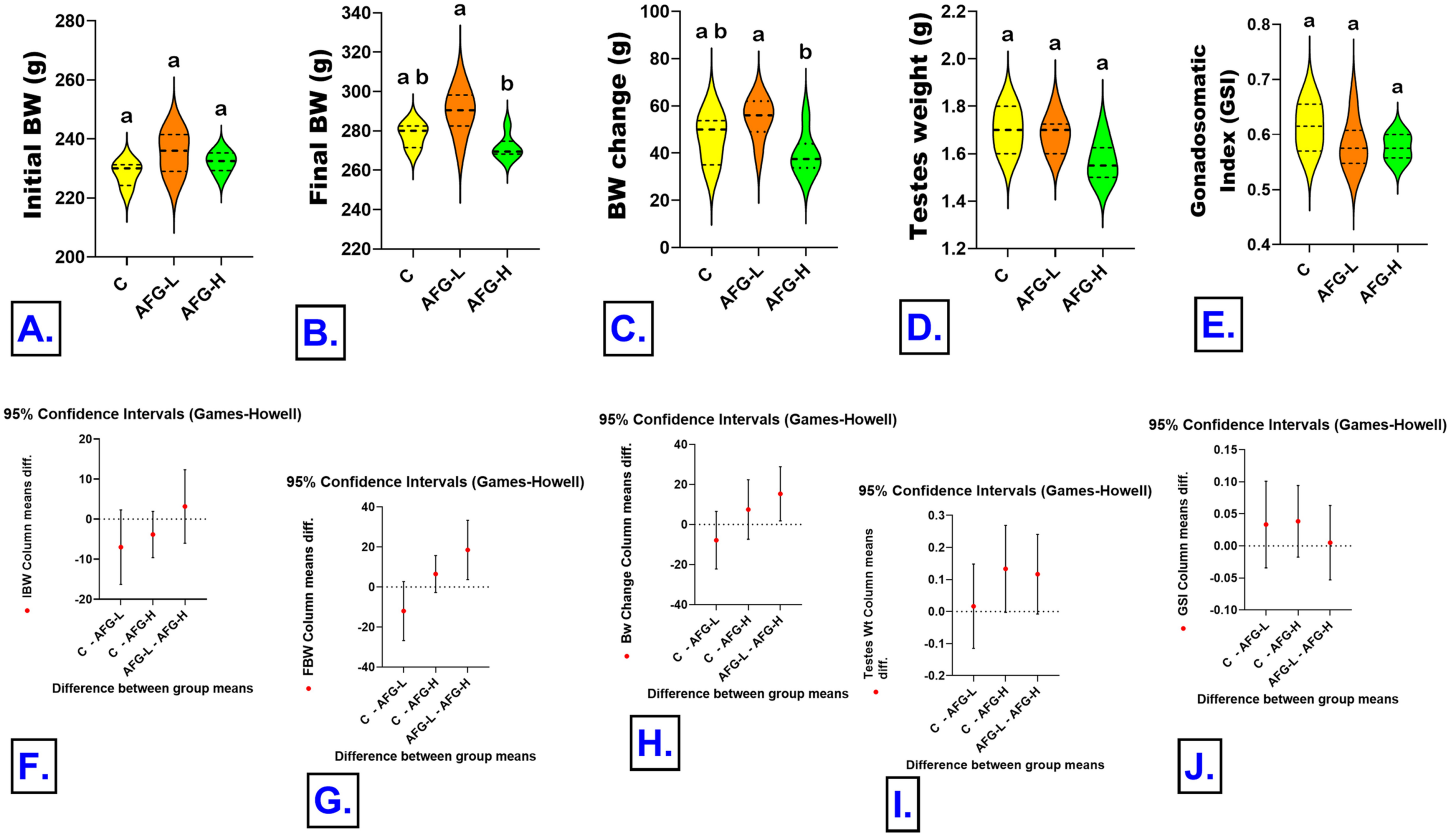
Effects of *A. fragrantissima* extract oral administration for 30 days on final body weight, relative testes weight of male Sprague Dawley rats. Group differences were analyzed using Welch’s ANOVA followed by Games–Howell multiple-comparison tests (*n* = 6 samples/group). Data are presented as violin plots showing distribution density with mean and interquartile range. Plots carrying different superscript letters indicate a significant difference between groups (*p* < 0.05). **(A)** Initial body weight (IBW; g), **(B)** Final body weight (FBW; g), **(C)** Body weight change (g), **(D)** Testes weight (g), **(E)** Gonado-somatic index (GSI), **(F–J)** Error bars represent pairwise comparisons with 95% confidence intervals (CI 95%) for the mean differences (*p* < 0.05).

Results are shown in Figure 1. Welch ANOVA revealed that the IBW did not differ significantly between the experimental groups, W (2, 9.43) = 2.73, *p* = 0.116. No significant difference between the AFG-L (235.33 ± 2.82) and the C (228.33 ± 1.61; 7.00, 95% CI [−2.30, 16.30], *p* = 0.140), between the AFG-H (232.33 ± 1.35; 3.83, 95% CI [−1.95, 9.61], *p* = 0.212) and the C, and between the AFG-L and AFG-H groups (3.17, 95% CI [−6.00, 12.33], *p* = 0.593). FBW differed significantly among the groups, W (2, 9.51) = 6.32, *p* = 0.018. The FBW significantly increased in AFG-L (290.00 ± 4.52) than the AFG-H (271.50 ± 2.43; 18.50, 95% CI [3.69, 33.31], *p* = 0.018), while, there were no significant differences between the AFG-L (290.00 ± 4.25) and the C groups (278.00 ± 2.32; 12.00, 95% CI [−2.75, 26.75], *p* = 0.108) or between the AFG-H (271.50 ± 2.43) and the C groups (6.50, 95% CI [−2.72, 15.72], *p* = 0.180). BW change differed significantly among the groups, W (2, 9.94) = 4.55, *p* = 0.040. The BW change significantly increased in AFG-L (54.67 ± 3.33) than the AFG-H (39.33 ± 3.63) group (15.33, 95% CI [1.81, 28.86], *p* = 0.027), while, there were no significant differences between the AFG-L (54.67 ± 3.33) and the C groups (46.38 ± 4.01; 7.83, 95% CI [−6.54, 22.21], *p* = 0.332) or between the AFG-H (39.33 ± 3.63) and the C groups (15.33, 95% CI [1.81, 28.86], *p* = 0.027). The testicular weight differed significantly among groups, W (2, 9.95) = 4.36, *p* = 0.044. The difference between the AFG-L (1.68 ± 0.03) and the C groups (1.70 ± 0.04; 0.017, 95% CI [−0.11, 0.15], *p* = 0.935), between the AFG-H (1.57 ± 0.03) and the C groups (1.70 ± 0.4; 0.13, 95% CI [−0.002, 0.27], *p* = 0.054), or between the AFG-L and AFG-H groups (0.12, 95% CI [−0.008, 0.24], *p* = 0.066). The GSI did not differ significantly between groups, W (2, 9.22) = 1.56, *p* = 0.261. The difference between the AFG-L (0.58 ± 0.02) and the C groups (0.61 ± 0.02; 0.030, 95% CI [−0.038, 0.098], *p* = 0.468), between the AFG-H (0.57 ± 0.01) and the C groups (0.61 ± 0.2; 0.035, 95% CI [−0.019, 0.090], *p* = 0.224), or between the AFG-L and AFG-H groups (0.005, 95% CI [−0.065, 0.056], *p* = 0.972).

**Figure 2:**
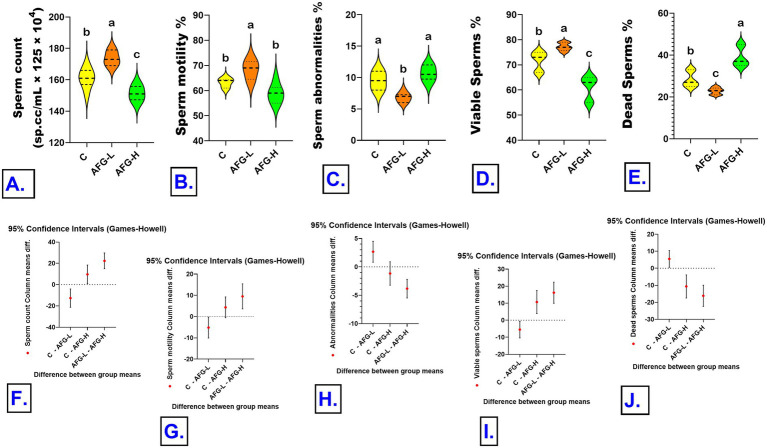
Effects of *A. fragrantissima* extract oral administration for 30 days on final body weight, relative testes weight of male Sprague Dawley rats. Group differences were analyzed using Welch’s ANOVA followed by Games–Howell multiple-comparison tests (*n* = 6 samples/group). Data are presented as violin plots showing distribution density with mean and interquartile range. Plots carrying different superscript letters indicate a significant difference between groups (*p* < 0.05). **(A)** Sperm count (sp. cc/mL × 125 × 10^6^), **(B)** Sperm motility (%), **(C)** Sperm abnormalities (%), **(D)** Viable sperms (%), **(E)** Dead sperms (%), **(F–J)** Error bars represent pairwise comparisons are reported with 95% confidence intervals (CI 95%) for the mean differences (*p* < 0.05).

Results of Welch ANOVA revealed that the sperm count differed significantly between the groups, W (2, 9.87) = 23.37, *p* < 0.001. Sperm count increased significantly in the AFG-L (173.67 ± 1.84; 12.67, 95% CI [4.09, 21.24], *p* = 0.006) and in the AFG-H (151.33 ± 1.98; 9.67, 95% CI [0.91, 18.42], *p* = 0.032) than in the C group (161.00 ± 2.48). Sperm count significantly increased in the AFG-L group than the AFG-H group (22.33, 95% CI [14.93, 29.74], *p* < 0.001). Sperm motility differed significantly among the groups, W (2, 8.69) = 9.15, *p* = 0.007. The motility significantly increased in AFG-L (68.33 ± 1.52) than in AFG-H (58.83 ± 1.51; 9.50, 95% CI [3.62, 15.38], *p* = 0.003), and significantly than the C groups (63.17 ± 0.70; 5.17, 95% CI [0.24, 10.09], *p* = 0.041). No significant difference between the AFG-H and the C groups (4.33, 95% CI [−0.57, 9.24], *p* = 0.080). Abnormal sperms % differed significantly among the groups W (2, 9.28) = 23.33, *p* < 0.001. The sperm abnormalities significantly decreased in AFG-L (6.83 ± 0.31; 2.67, 95% CI [0.82, 4.51], *p* = 0.008) than the C groups (9.50 ± 0.56) and the AFG-H (10.67 ± 0.49; 3.83, 95% CI [2.18, 5.48], *p* < 0.001), while, there were no significant differences between the AFG-H (10.67 ± 0.49) and the C groups (9.50 ± 0.56; 1.17, 95% CI [−0.89, 3.23], *p* = 0.308); results are shown in Figure 2.

The live and dead sperms % differed significantly among groups, W (2, 8.23) = 31.58, *p* < 0.001. The live sperm % increased significantly in the AFG-L (77.17 ± 0.65) than the C groups (71.67 ± 1.52; 5.50, 95% CI [0.59, 10.41], *p* = 0.031) and the AFG-H (1.57 ± 0.03; 61.00 ± 1.93; 16.17, 95% CI [9.95, 22.39], *p* < 0.001). Live sperm % significantly decreased in the AFG-H groups (61.00 ± 1.93) than the C groups (71.67 ± 1.52; 10.67, 95% CI [3.87, 17.47], *p* = 0.004). The dead sperm % decreased significantly in the AFG-L (22.83 ± 0.65) than the C groups (28.33 ± 1.25; 5.50, 95% CI [0.59, 10.41], *p* = 0.031) and the AFG-H (39.00 ± 1.93; 16.17, 95% CI [9.95, 22.39], *p* < 0.001). Dead sperm % significantly increased in the AFG-H groups than the C groups (10.67, 95% CI [3.87, 17.47], *p* = 0.004); results are shown in Figure 2.

### 3.3 Effects of *Achillea Fragrantissima* extract administration on hematology and phagocytosis assay

Results of the differential leukocytic counts are displayed in Table 3. WBC count differed significantly among the groups, W (2, 8.25) = 54.98, *p* < 0.001. WBC count significantly increased in the AFG-H (11.06 ± 0.33) than the C (7.20 ± 0.13; 3.86, 95% CI [2.79, 4.93], *p* < 0.001) and the AFG-L (7.85 ± 0.32; 3.21, 95% CI [1.94, 4.48], *p* <0.001) groups. There were no significant differences between the AFG-L and the C groups (0.65, 95% CI [−0.39, 1.70], *p* = 0.221).

**Table 3:** Effect of *A. fragrantissima* extract oral administration on hematology- and phagocytosis assay-related indices of adult male Sprague Dawley rats for 4 weeks.

Hematology-related indices	W (df1, df2)	*p*-value	C	AFG-L	AFG-H	*Post-hoc* Games Howell (Significant pairs)
WBC (10^3^/ml^3^)	54.98 (2, 8.25)	0.000	7.20 ± 0.13 ^b^	7.85 ± 0.32 ^b^	11.06 ± 0.33 ^a^	2 > 1 (0.65, 95% CI [−0.39, 1.70], *p* = 0.221); 3 > 1 (3.86, 95% CI [2.79, 4.93], *p* < 0.001); 3 > 2 (3.21, 95% CI [1.94, 4.48], *p* <0.001)
LYM (10^3^/ml^3^)	61.04 (2, 9.65)	0.007	4.65 ± 0.18 ^b^	5.00 ± 0.25 ^b^	8.14 ± 0.28 ^a^	2 > 1 (0.35, 95% CI [−0.58, 1.29], *p* = 0.558); 3 > 1 (3.49, 95% CI [2.61, 4.36], *p* < 0.001); 3 > 2 (3.14, 95% CI [2.10, 4.17], *p* < 0.001)
MON (10^3^/ml^3^)	1.15 (2, 8.18)	0.362	1.13 ± 0.07 ^a^	1.34 ± 0.15 ^a^	1.41 ± 0.25 ^a^	2 > 1 (0.22, 95% CI [−0.28, 0.71], *p* = 0.442); 3 > 1 (0.28, 95% CI [−0.55, 1.12], *p* = 0.577); 3 > 2 (0.063, 95% CI [−0.79, 0.92], *p* = 0.976)
GRAN (10^3^/ml^3^)	0.319 (2, 7.87)	0.736	1.43 ± 0.05 ^a^	1.51 ± 0.17 ^a^	1.52 ± 0.12 ^a^	2 > 1 (0.082, 95% CI [−0.48, 0.64], *p* = 0.893); 3 > 1 (0.092, 95% CI [−0.28, 0.47], *p* = 0.753); 2 > 3 (0.010, 95% CI [−0.58, 0.60], *p* = 0.999)
LYM %	12.95 (2, 8.85)	0.002	64.47 ± 1.52 ^b^	63.97 ± 3.51 ^ab^	73.59 ± 1.04 ^a^	2 > 1 (0.50, 95% CI [−10.85, 11.85], *p* = 0.991); 3 > 1 (9.12, 95% CI [3.95, 14.29], *p* = 0.002); 3 > 2 (9.62, 95% CI [−1.69, 20.93], *p* = 0.088)
MON %	1.25 (2, 9.40)	0.330	15.69 ± 1.15 ^a^	16.99 ± 1.67 ^a^	12.63 ± 2.11 ^a^	2 > 1 (1.30, 95% CI [−4.39, 6.99], *p* = 0.803); 1 > 3 (3.07, 95% CI [−3.85, 9.98], *p* = 0.447); 2 > 3 (4.37, 95% CI [−3.08, 11.81], *p* = 0.284)
GRAN %	10.12 (2, 9.02)	0.005	19.82 ± 0.73 ^a^	19.02 ± 1.86 ^ab^	13.77 ± 1.08 ^b^	1 > 2 (0.79, 95% CI [−5.20, 6.79], *p* = 0.917); 1 > 3 (6.05, 95% CI [2.38, 9.71], *p* = 0.003); 2 > 3 (5.25, 95% CI [−0.89, 11.39], *p* = 0.092)
RBCs (10^6^/ml^3^)	2.00 (2, 8.91)	0.192	7.67 ± 0.20 ^a^	7.64 ± 0.08 ^a^	7.31 ± 0.14 ^a^	1 > 2 (0.03, 95% CI [−0.62, 0.68], *p* = 0.990); 1 > 3 (0.36, 95% CI [−0.33, 1.04], *p* = 0.352); 2 > 3 (0.33, 95% CI [−0.14, 0.79], *p* = 0.173)
Hb (g/dl)	1.82 (2, 9.45)	0.214	14.00 ± 0.36 ^a^	14.23 ± 0.23 ^a^	13.65 ± 0.18 ^a^	2 > 1 (0.23, 95% CI [−0.99, 1.46], *p* = 0.855); 1 > 3 (0.35, 95% CI [−0.84, 1.54], *p* = 0.683); 2 > 3 (0.58, 95% CI [−0.24, 1.41], *p* = 0.178)
PCV %	0.943 (2, 7.30)	0.432	44.23 ± 0.64 ^a^	43.27 ± 0.18 ^a^	43.12 ± 1.14 ^a^	1 > 2 (0.96, 95% CI [−1.13, 3.04], *p* = 0.391); 1 > 3 (1.11, 95% CI [−2.64, 4.86], *p* = 0.686); 2 > 3 (0.16, 95% CI [−3.53, 3.84], *p* = 0.990)
MCV (fl)	1.46 (2, 9.34)	0.281	55.67 ± 0.55 ^a^	56.50 ± 0.76 ^a^	57.83 ± 1.13 ^a^	2 > 1 (0.83, 95% CI [−1.79, 3.47], *p* = 0.665); 3 > 1 (2.17, 95% CI [−1.53, 5.86], *p* = 0.265); 3 > 2 (1.33, 95% CI [−2.51, 5.18], *p* = 0.611)
MCH %	1.09 (2, 8.89)	0.375	18.06 ± 0.36 ^a^	18.65 ± 0.28 ^a^	18.68 ± 0.16 ^a^	2 > 1 (0.58, 95% CI [−0.71, 1.88], *p* = 0.454); 3 > 1 (0.61, 95% CI [−0.58, 1.81], *p* = 0.339); 3 > 2 (0.030, 95% CI [−0.91, 0.97], *p* = 0.995)
MCHC %	3.80 (2, 8.73)	0.065	33.20 ± 0.22 ^a^	32.93 ± 0.51 ^a^	31.73 ± 0.45 ^a^	2 > 1 (0.27, 95% CI [−1.39, 1.93], *p* = 0.886); 1 > 3 (1.46, 95% CI [−0.03, 2.95], *p* = 0.054); 2 > 3 (1.19, 95% CI [−0.69, 3.09], *p* = 0.241)
Ph%	20.12 (2, 9.97)	0.000	9.00 ± 0.63 ^b^	14.50 ± 0.56 ^a^	12.66 ± 0.55 ^a^	2 > 1 (5.50, 95% CI [3.17, 7.83], *p* < 0.001); 3 > 1 (3.67, 95% CI [1.35, 5.98], *p* = 0.004); 2 > 3 (1.83, 95% CI [−0.34, 4.01], *p* = 0.100)
PhI	152.42 (2, 9.31)	0.000	1.71 ± 0.03 ^c^	2.56 ± 0.06 ^a^	2.26 ± 0.02 ^b^	2 > 1 (0.85, 95% CI [0.65, 1.04], *p* < 0.001); 3 > 1 (0.55, 95% CI [0.45, 0.65], *p* < 0.001); 2 > 3 (0.29, 95% CI [0.11, 0.49], *p* = 0.007)

Values are mean ± SEM of 6 animals per experimental group. Welch One-Way Analysis of Variance (ANOVA) followed by the Games-Howell test. Groups carrying the same superscript letters indicate no significant differences at the *p* < 0.05 level. WBCs, white blood cells; LYM, lymphocytes; MON, monocytes; GRAN, granulocytes; RBCs, red blood cells; Hb, hemoglobin; PCV, packed cell volume; MCV, mean corpuscular volume; MCH, mean corpuscular hemoglobin; MCHC, mean corpuscular hemoglobin concentration; Ph%, phagocytic %; PhI, phagocytic index.

Lymphocyte count differed significantly across the groups, W (2, 9.65) = 61.04, *p* = 0.007. Lymphocyte count significantly increased in the AFG-H (8.14 ± 0.28) than the C (4.65 ± 0.18; 3.49, 95% CI [2.61, 4.36], *p* < 0.001) and the AFG-L (5.00 ± 0.25; 3.14, 95% CI [2.10, 4.17], *p* < 0.001) groups. There were no significant differences between the AFG-L and the C groups (0.35, 95% CI [−0.58, 1.29], *p* = 0.558). There were no significant differences between groups in the monocyte count, W (2, 8.18) = 1.15, *p* = 0.362, granuocytes count W (2, 7.87) = 0.319, *p* = 0.736, and monocytes % W (2, 9.40) = 1.25, *p* = 0.330. Results are shown in Table 3. Lymphocytes % differed significantly between the groups, W (2, 8.85) = 12.95, *p* = 0.002. Lymphocytes % significantly increased in the AFG-H (73.59 ± 1.04) than the C (64.47 ± 1.52; 9.12, 95% CI [3.95, 14.29], *p* = 0.002). There were no significant differences between the AFG-L (63.97 ± 3.51) and the C groups (64.47 ± 1.52; 0.50, 95% CI [−10.85, 11.85], *p* = 0.991) or between the AFG-L and the AFG-H (9.62, 95% CI [−1.69, 20.93], *p* = 0.088) groups; results are shown in Table 3.

Granulocytes % differed significantly between the groups, W (2, 9.02) = 10.12, *p* = 0.005. Granulocytes % significantly decreased in the AFG-H (13.77 ± 1.08) than the C (19.82 ± 0.73; 6.05, 95% CI [2.38, 9.71], *p* = 0.003). There were no significant differences between the AFG-L (19.02 ± 1.86) and the C groups (19.82 ± 0.73; 0.79, 95% CI [−5.20, 6.79], *p* = 0.917) or between the AFG-L and the AFG-H (5.25, 95% CI [−0.89, 11.39], *p* = 0.092) groups.

Results of the erythrogram are presented in Table 3. Results of Welch ANOVA revealed that the RBCs count, W (2, 8.91) = 2.00, *p* = 0.192, Hb content, W (2, 9.45) = 1.82, *p* = 0.214, PCV, W (2, 7.30) = 0.943, *p* = 0.432, MCV, W (2, 9.34) = 1.46, *p* = 0.281, MCH %, W (2, 8.89) = 1.09, *p* = 0.375, and MCHC %, W (2, 8.73) = 3.80, *p* = 0.065, % did not differ significantly between the groups. Results of Welch ANOVA revealed that the Ph% differed significantly across groups, W (2, 9.97) = 20.12, *p* < 0.001. Ph% increased significantly in the AFG-L (14.50 ± 0.56; 5.50, 95% CI [3.17, 7.83], *p* < 0.001) and in the AFG-H (12.66 ± 0.55; 3.67, 95% CI [1.35, 5.98], *p* = 0.004) than the C group (9.00 ± 0.63). No significant differences between the AFG-L group and the AFG-H group (1.83, 95% CI [−0.34, 4.01], *p* = 0.100) were observed. PhI differed significantly between groups, W (2, 9.31) = 152.42, *p* < 0.001. PhI increased significantly in the AFG-L (2.56 ± 0.06; 0.85, 95% CI [0.65, 1.04], *p* < 0.001) and in the AFG-H (2.26 ± 0.02; 0.55, 95% CI [0.45, 0.65], *p* < 0.001) than in the C group (1.71 ± 0.06). The PhI differed significantly between the AFG-L group and the AFG-H group (0.29, 95% CI [0.11, 0.49], *p* = 0.007); results are presented in Table 3.

### 3.4 Effects of *Achillea Fragrantissima* extract administration on serum biochemical and measurements and lipid profile

The obtained results, shown in Table 4, indicate that Welch ANOVA revealed a significant difference in TEST across groups, W (2, 9.74) = 12.58, *p* = 0.002. TEST increased significantly in the AFG-L (6.83 ± 0.24; 2.03, 95% CI [0.94, 3.11], *p* < 0.001) and in the AFG-H (6.30 ± 0.36; 1.49, 95% CI [0.19, 2.80], *p* = 0.026) than in the C group (4.81 ± 0.31). No significant differences between the AFG-L group and the AFG-H group (0.53, 95% CI [−0.69, 1.75], *p* = 0.474) were observed. FSH differed significantly between groups, W (2, 7.89) = 15.11, *p* = 0.002. FSH increased significantly in the AFG-L (4.69 ± 0.20; 1.19, 95% CI [0.53, 1.85], *p* = 0.003) than in the C group (4.81 ± 0.31). No significant differences were observed between the AFG-H (4.20 ± 0.29) and the C groups (4.81 ± 0.31; 0.69, 95% CI [−0.24, 1.64], *p* = 0.134) or between the AFG-L and the AFG-H groups (0.49, 95% CI [−0.50, 1.49], *p* = 0.390). LH differed significantly between groups, W (2, 9.46) = 15.16, *p* = 0.001. LH increased significantly in the AFG-L (32.45 ± 0.68; 6.73, 95% CI [3.43, 10.02], *p* = 0.001) than in the C group (25.72 ± 0.96). No significant differences observed between the AFG-H (29.78 ± 1.22) and the C groups (25.72 ± 0.96; 4.06, 95% CI [−0.25, 8.36], *p* = 0.065) or between the AFG-L and the AFG-H groups (2.67, 95% CI [−1.35, 6.68], *p* = 0.200). Serum E2 differed significantly among groups, W (2, 9.99) = 27.39, *p* < 0.001. E2 increased significantly in the AFG-L (28.19 ± 0.67; 7.25, 95% CI [4.62, 9.88], *p* < 0.001) and in the AFG-H (25.64 ± 0.69; 4.69, 95% CI [2.01, 7.36], *p* = 0.002) than in the C group (20.95 ± 0.68). No significant differences between the AFG-L group and the AFG-H group (2.56, 95% CI [−0.094, 5.22], *p* = 0.059) were observed. Welch ANOVA revealed a significant difference in TNF-*α* across groups, W (2, 9.14) = 25.39, *p* < 0.001. TNF-α decreased significantly in the AFG-L (119.6 ± 2.32; 2.03, 95% CI [0.94, 3.11], *p* < 0.001) than the C group (134.8 ± 2.74). TNF-α increased significantly in the AFG-H (170.7 ± 7.18) than in the C group (35.84, 95% CI [12.69, 58.98], *p* = 0.007) and in the AFG-L group (51.09, 95% CI [27.96, 74.21], *p* < 0.001). IL-6 differed significantly between groups, W (2, 9.93) = 107.50, *p* < 0.001. IL-6 decreased significantly in the AFG-L (173.5 ± 3.42) than in the C group (184.1 ± 2.25; 10.66, 95% CI [2.00, 19.32], *p* = 0.018). IL-6 increased significantly in the AFG-H (225.00 ± 2.69) than in the C group (40.83, 95% CI [−31.19, 50.46], *p* < 0.001) and than the AFG-L group (51.49, 95% CI [41.83, 61.15], *p* < 0.001); results are presented in Table 4.

**Table 4:** Effect of *A. fragrantissima* extract oral administration on hematology- and phagocytosis assay-related indices of adult male Sprague Dawley rats for 4 weeks.

Parameters	W (df1, df2)	*p*-value	C	AFG-L	AFG-H	*Post-hoc* Games Howell (Significant pairs)
TEST ng/mL	12.58 (2, 9.74)	0.002	4.81 ± 0.31 ^b^	6.83 ± 0.24 ^a^	6.30 ± 0.36^a^	2 > 1 (2.03, 95% CI [0.94, 3.11], *p* < 0.001); 3 > 1 (1.49, 95% CI [0.19, 2.80], *p* = 0.026); 2 > 3 (0.53, 95% CI [−0.69, 1.75], *p* = 0.474)
FSH IU/mL	15.11 (2, 7.89)	0.002	3.50 ± 0.08 ^b^	4.69 ± 0.20 ^a^	4.20 ± 0.29 ^ab^	2 > 1 (1.19, 95% CI [0.53, 1.85], *p* = 0.003); 3 > 1 (0.69, 95% CI [−0.24, 1.64], *p* = 0.134); 2 > 3 (0.49, 95% CI [−0.50, 1.49], *p* = 0.390)
LH IU/mL	15.16 (2, 9.46)	0.001	25.72 ± 0.96 ^b^	32.45 ± 0.68 ^a^	29.78 ± 1.22 ^ab^	2 > 1 (6.73, 95% CI [3.43, 10.02], *p* = 0.001); 3 > 1 (4.06, 95% CI [−0.25, 8.36], *p* = 0.065); 2 > 3 (2.67, 95% CI [−1.35, 6.68], *p* = 0.200)
E2 pg/mL	27.39 (2, 9.99)	0.000	20.95 ± 0.68 ^b^	28.19 ± 0.67 ^a^	25.64 ± 0.69 ^a^	2 > 1 (7.25, 95% CI [4.62, 9.88], *p* < 0.001); 3 > 1 (4.69, 95% CI [2.01, 7.36], *p* = 0.002); 2 > 3 (2.56, 95% CI [−0.094, 5.22], *p* = 0.059)
Tnf-a pg/mL	25.39 (2, 9.14)	0.000	134.8 ± 2.74 ^b^	119.6 ± 2.32 ^c^	170.7 ± 7.18 ^a^	2 < 1 (15.24, 95% CI [5.35, 25.14], *p* = 0.005); 3 > 1 (35.84, 95% CI [12.69, 58.98], *p* = 0.007); 3 > 2 (51.09, 95% CI [27.96, 74.21], *p* < 0.001)
IL-6 pg/mL	107.50 (2, 9.93)	0.000	184.1 ± 2.25 ^b^	173.5 ± 3.42 ^c^	225.0 ± 2.69 ^a^	2 < 1 (10.66, 95% CI [2.00, 19.32], *p* = 0.018); 3 > 1 (40.83, 95% CI [−31.19, 50.46], *p* < 0.001); 3 > 2 (51.49, 95% CI [41.83, 61.15], *p* < 0.001)
T. C mg/dl	209.29 (2, 9.43)	0.000	242.7 ± 3.73 ^a^	126.1 ± 4.11 ^c^	167.0 ± 7.97 ^b^	1 > 2 (116.59, 95% CI [101.34, 131.84], *p* < 0.001); 1 > 3 (75.72, 95% CI [49.87, 101.57], *p* < 0.001); 3 > 2 (40.87, 95% CI [14.86, 66.89], *p* = 0.005)
T. Gs mg/dl	14.27 (2, 9.22)	0.002	147.8 ± 3.32 ^a^	106.6 ± 7.43 ^b^	128.57 ± 4.69 ^b^	1 > 2 (41.23, 95% CI [17.18, 65.29], *p* = 0.004); 1 > 3 (19.30, 95% CI [3.24, 35.36], *p* = 0.021); 3 > 2 (21.93, 95% CI [−2.92, 46.79], *p* = 0.083)
HDL mg/dl	25.38 (2, 8.41)	0.000	50.63± 0.23 ^a^	48.36 ± 0.45 ^b^	45.41 ± 0.78 ^c^	2 < 1 (2.27, 95% CI [0.80, 3.73], *p* = 0.006); 3 < 1 (5.22, 95% CI [2.71, 7.72], *p* = 0.002); 2 > 3 (2.95, 95% CI [0.39, 5.51], *p* = 0.027)
LDL mg/dl	329.78 (2, 8.79)	0.000	162.56± 3.30 ^a^	56.48 ± 2.23 ^c^	95.92 ± 8.08 ^b^	1 > 2 (106.08, 95% CI [94.90, 117.25], *p* < 0.001); 1 > 3 (66.64, 95% CI [40.67, 92.61], *p* < 0.001); 3 > 2 (39.44, 95% CI [13.49, 65.38], *p* = 0.009)
VLDL mg/dl	14.29 (2, 9.21)	0.002	29.57± 0.66 ^a^	21.33± 1.49 ^b^	25.71± 0.94 ^b^	2 < 1 (8.24, 95% CI [3.43, 13.05], *p* = 0.004); 3 < 1 (3.86, 95% CI [0.65, 7.07], *p* = 0.021); 3 > 2 (4.38, 95% CI [−0.59, 9.35], *p* = 0.083)

Values are mean ± SEM of 6 animals per experimental group. Welch One-Way Analysis of Variance (ANOVA) followed by the Games-Howell test. TEST, testosterone; FSH, follicle-stimulating hormone; LH, luteinizing hormone; E2, estradiol; TC, total cholesterol; TGs, triglycerides; HDL, high-density lipoproteins; LDL, low-density lipoproteins; VLDL, very low-density lipoproteins. Groups carrying the same superscript letters indicate no significant differences at the *p* < 0.05 level.

Serum TC differed significantly between groups, W (2, 9.43) = 209.29, *p* < 0.001. TC decreased significantly in the AFG-L (126.1 ± 4.11; 116.59, 95% CI [101.34, 131.84], *p* < 0.001) and in the AFG-H (167.00 ± 7.97; 75.72, 95% CI [49.87, 101.57], *p* < 0.001) than in the C group (242.7 ± 3.73). TC decreased significantly in the AFG-L than the AFG-H group (40.87, 95% CI [14.86, 66.89], *p* = 0.005). Serum TGs differed significantly between groups, W (2, 9.22) = 14.27, *p* = 0.002. TGs decreased significantly in the AFG-L (106.6 ± 7.73; 41.23, 95% CI [17.18, 65.29], *p* = 0.004) and in the AFG-H (128.57 ± 4.69; 19.30, 95% CI [3.24, 35.36], *p* = 0.021) than in the C group (147.8 ± 3.32). No significant differences between the AFG-L and the AFG-H groups (21.93, 95% CI [−2.92, 46.79], *p* = 0.083) were observed. HDL differed significantly between groups, W (2, 8.41) = 25.38, *p* < 0.001. HDL decreased significantly in the AFG-L (48.36 ± 0.45; 2.27, 95% CI [0.80, 3.73], *p* = 0.006) and in the AFG-H (45.41 ± 0.78; 5.22, 95% CI [2.71, 7.72], *p* = 0.002) than in the C group (50.63 ± 0.23). HDL decreased significantly in the AFG-H than the AFG-L group (2.95, 95% CI [0.39, 5.51], *p* = 0.027). LDL differed significantly between groups, W (2, 8.79) = 329.78, *p* < 0.001. LDL decreased significantly in the AFG-L (56.48 ± 2.23; 106.08, 95% CI [94.90, 117.25], *p* < 0.001) and in the AFG-H (95.92 ± 8.08; 66.64, 95% CI [40.67, 92.61], *p* < 0.001) than in the C group (162.56 ± 3.30). LDL decreased significantly in the AFG-L than the AFG-H group (39.44, 95% CI [13.49, 65.38], *p* = 0.009). VLDL differed significantly between groups, W (2, 9.21) = 14.29, *p* = 0.002. VLDL decreased significantly in the AFG-L (21.33 ± 1.49; 8.24, 95% CI [3.43, 13.05], *p* = 0.004) and in the AFG-H (25.71 ± 0.94; 3.86, 95% CI [0.65, 7.07], *p* = 0.021) than in the C group (29.57 ± 0.66). No significant differences between the AFG-L and the AFG-H groups; 3 < 1; 3 > 2 (4.38, 95% CI [−0.59, 9.35], *p* = 0.083) were observed.; results are presented in Table 4.

### 3.5 Effects of *Achillea Fragrantissima* extract on oxidative stress indicators in spleen and testes

Results regarding the effects of AFG administration on testicular levels of TAC, GPx, SOD, MDA, PCO, 8OH2dG, and LDH are presented in Table 5. Welch ANOVA revealed a significant difference in testicular TAC across groups, W (2, 9.99) = 25.06, *p* < 0.001. TAC increased significantly in the AFG-L (5.47 ± 0.19; 1.99, 95% CI [1.23, 2.75], *p* < 0.001) and in the AFG-H groups (4.27 ± 0.19; 0.79, 95% CI [−0.03, 1.56], *p* = 0.043) than in the C group (3.47 ± 0.20). TAC significantly increased in the AFG-L group than the AFG-H group (1.20, 95% CI [0.46, 1.93], *p* = 0.003). Testicular SOD activity levels differed significantly across groups, W (2, 8.79) = 41.46, *p* < 0.001. SOD increased significantly in the AFG-L group (121.60 ± 6.17; 59.47, 95% CI [39.60, 79.34], *p* < 0.001) and in the AFG-H group (79.85 ± 3.65; 17.72, 95% CI [5.60, 29.84], *p* = 0.008) than in the C group (62.13 ± 2.20). SOD activity significantly increased in the AFG-L group than the AFG-H group (41.75, 95% CI [21.33, 62.18], *p* < 0.001). Testicular GPx activity levels differed significantly across groups, W (2, 8.29) = 76.38, *p* < 0.001. GPx increased significantly in the AFG-L group (110.55 ± 3.03; 41.77, 95% CI [31.99, 51.54], *p* < 0.001) and in the AFG-H group (80.59 ± 3.30; 11.82, 95% CI [1.18, 22.45], *p* = 0.033) than the C group (68.78 ± 1.25). GPx significantly increased in the AFG-L group than the AFG-H group (29.95, 95% CI [17.65, 42.24], *p* < 0.001). Testicular MDA levels differed significantly across groups, W (2, 8.78) = 56.56, *p* < 0.001. MDA decreased significantly in the AFG-L group (1.24 ± 0.04; 1.45, 95% CI [0.92, 1.97], *p* < 0.001) and in the AFG-H group (1.78 ± 0.06; 0.91, 95% CI [0.38, 1.43], *p* = 0.004) than the C group (2.69 ± 0.16). MDA levels were significantly reduced in the AFG-L group than the AFG-H group (0.54, 95% CI [0.35, 0.73], *p* < 0.001). Testicular PCO levels differed significantly across groups, W (2, 8.31) = 353.86, *p* < 0.001. PCO levels decreased significantly in the AFG-L group (2.70 ± 0.05; 3.16, 95% CI [2.82, 3.50], *p* < 0.001) and in the AFG-H group (4.13 ± 0.16; 1.73, 95% CI [1.20, 2.26], *p* < 0.001) than in the C group (5.86 ± 0.11). PCO levels significantly decreased in the AFG-L group than the AFG-H group (1.43, 95% CI [0.92, 1.94], *p* < 0.001). Testicular 8OH2dG levels differed significantly across groups, W (2, 7.65) = 73.87, *p* < 0.001. 8OH2dG decreased significantly in the AFG-L group (2.26 ± 0.07; 2.89, 95% CI [2.18, 3.60], *p* < 0.001) and in the AFG-H group (3.43 ± 0.32; 1.71, 95% CI [0.62, 2.81], *p* = 0.005) than the C group (5.15 ± 0.22). 8OH2dG levels significantly decreased in the AFG-L group than the AFG-H group (1.18, 95% CI [0.14, 2.22], *p* = 0.031). Testicular LDH levels differed significantly across groups, W (2, 9.19) = 63.82, *p* < 0.001. LDH levels decreased significantly in the AFG-L group (14.05 ± 0.44; 4.71, 95% CI [3.13, 6.28], *p* < 0.001) and in the AFG-H group (25.89 ± 1.06; 7.14, 95% CI [3.74, 10.55], *p* < 0.001) than the C group (18.75 ± 0.37). LDH levels significantly decreased in the AFG-L group than the AFG-H group (11.85, 95% CI [8.44, 15.26], *p* < 0.001).

**Table 5:** Effect of *A. fragrantissima* extract oral administration on oxidative stress-related indices of adult male Sprague Dawley rats for 4 weeks.

Oxidative stress-related indices		W (df1, df2)	*p*-value	C	AFG-L	AFG-H	*Post-hoc* Games Howell (Significant pairs)
Spleen	TAC (ng/mg tissue)	153.14 (2, 8.96)	0.000	3.71 ± 0.12 ^c^	9.44 ± 0.45 ^a^	6.81 ± 0.16 ^b^	2 > 1 (5.73, 95% CI [4.28, 7.17], *p* < 0.001); 3 > 1 (3.09, 95% CI [2.53, 3.66], *p* < 0.001); 2 > 3 (2.63, 95% CI [1.19, 4.08], *p* = 0.003)
	SOD (U/mg tissue)	153.89 (2, 9.82)	0.000	82.21± 1.69 ^c^	130.34 ± 2.08 ^a^	108.32 ± 2.33 ^b^	2 > 1 (48.13, 95% CI [40.73, 55.53], *p* < 0.001); 3 > 1 (26.11, 95% CI [−18.08, 34.14], *p* < 0.001); 2 > 3 (22.02, 95% CI [13.44, 30.60], *p* < 0.001)
	GPx (U/mg tissue)	142.44 (2, 9.58)	0.000	81.93± 2.53 ^c^	132.88 ± 1.55 ^a^	111.08 ± 2.15 ^b^	2 > 1 (50.95, 95% CI [42.54, 59.37], *p* < 0.001); 3 > 1 (29.15, 95% CI [20.02, 38.29], *p* < 0.001); 2 > 3 (21.80, 95% CI [14.42, 29.18], *p* < 0.001)
	MDA (nmol/mg tissue)	39.45 (2, 9.57)	0.000	1.87 ± 0.06 ^a^	1.29 ± 0.03 ^c^	1.55± 0.04 ^b^	2 < 1 (0.58, 95% CI [0.39, 0.77], *p* < 0.001); 3 < 1 (0.32, 95% CI [0.13, 0.51], *p* = 0.004); 2 < 3 (0.26, 95% CI [0.13, 0.39], *p* < 0.001)
	PCO (ng/mg tissue)	234.43 (2, 9.17)	0.000	6.01 ± 0.09 ^a^	2.72 ± 0.13 ^c^	3.39 ± 0.19 ^b^	2 < 1 (3.29, 95% CI [2.86, 3.73], *p* < 0.001); 3 < 1 (2.62, 95% CI [1.99, 3.26], *p* < 0.001); 2 < 3 (0.67, 95% CI [0.01, 1.33], *p* = 0.047)
	8OH2dG (ng/mg tissue)	147.22 (2, 9.69)	0.000	6.96 ± 0.11 ^a^	3.74 ± 0.18 ^c^	4.84 ± 0.12 ^b^	2 < 1 (3.22, 95% CI [2.63, 3.80], *p* < 0.001); 3 < 1 (2.12, 95% CI [1.69, 2.55], *p* < 0.001); 2 < 3 (1.10, 95% CI [0.51, 1.69], *p* = 0.002)
Testes	TAC (ng/mg tissue)	25.06 (2, 9.99)	0.000	3.47 ± 0.20 ^c^	5.47 ± 0.19 ^a^	4.27 ± 0.19 ^b^	2 > 1 (1.99, 95% CI [1.23, 2.75], *p* < 0.001); 3 > 1 (0.79, 95% CI [−0.03, 1.56], *p* = 0.043); 2 > 3 (1.20, 95% CI [0.46, 1.93], *p* = 0.003)
	SOD (U/mg tissue)	41.46 (2, 8.79)	0.000	62.13± 2.20 ^c^	121.60 ± 6.17 ^a^	79.85± 3.65 ^b^	2 > 1 (59.47, 95% CI [39.60, 79.34], *p* < 0.001); 3 > 1 (17.72, 95% CI [5.60, 29.84], *p* = 0.008); 2 > 3 (41.75, 95% CI [21.33, 62.18], *p* < 0.001)
	GPx (U/mg tissue)	76.38 (2, 8.29)	0.000	68.78± 1.25 ^c^	110.55 ± 3.03 ^a^	80.59± 3.30 ^b^	2 > 1 (41.77, 95% CI [31.99, 51.54], *p* < 0.001); 3 > 1 (11.82, 95% CI [1.18, 22.45], *p* = 0.033); 2 > 3 (29.95, 95% CI [17.65, 42.24], *p* < 0.001)
	MDA (nmol/mgtissue)	56.56 (2, 8.78)	0.000	2.69 ± 0.16 ^a^	1.24 ± 0.04 ^c^	1.78 ± 0.06 ^b^	2 < 1 (1.45, 95% CI [0.92, 1.97], *p* < 0.001); 3 < 1 (0.91, 95% CI [0.38, 1.43], *p* = 0.004); 2 < 3 (0.54, 95% CI [0.35, 0.73], *p* < 0.001)
	PCO (ng/mg tissue)	353.86 (2, 8.31)	0.000	5.86 ± 0.11 ^a^	2.70 ± 0.05 ^c^	4.13 ± 0.16 ^b^	2 < 1 (3.16, 95% CI [2.82, 3.50], *p* < 0.001); 3 < 1 (1.73, 95% CI [1.20, 2.26], *p* < 0.001); 2 < 3 (1.43, 95% CI [0.92, 1.94], *p* < 0.001)
	8OH2dG (ng/mg tissue)	73.87 (2, 7.65)	0.000	5.15 ± 0.22 ^a^	2.26 ± 0.07 ^c^	3.43± 0.32 ^b^	2 < 1 (2.89, 95% CI [2.18, 3.60], *p* < 0.001); 3 < 1 (1.71, 95% CI [0.62, 2.81], *p* = 0.005); 2 < 3 (1.18, 95% CI [0.14, 2.22], *p* = 0.031)
	LDH (U/L)	63.82 (2, 9.19)	0.000	18.75 ± 0.37 ^b^	14.05 ± 0.44 ^c^	25.89 ± 1.06 ^a^	2 < 1 (4.71, 95% CI [3.13, 6.28], *p* < 0.001); 3 > 1 (7.14, 95% CI [3.74, 10.55], *p* < 0.001); 3 > 2 (11.85, 95% CI [8.44, 15.26], *p* < 0.001)

Values are mean ± SEM of 6 animals per experimental group. Welch One-Way Analysis of Variance (ANOVA) followed by the Games-Howell test. TAC, Total antioxidant capacity; SOD, Superoxide dismutase; GPx, Glutathione peroxidase; MDA, Malondialdehyde; PCO, Protein carbonyl; 8OH2dG, 8-hydroxy-2-deoxy-guanosine; LDH, Lactate dehydrogenase activity. Groups carrying the same superscript letters indicate no significant differences at the *p* < 0.05 level.

Results regarding the effects of AFG administration on levels of TAC, GPx, SOD, MDA, PCO, and 8OH2dG in the spleen of male rats are presented in Table 5. Welch ANOVA revealed a significant difference in splenic TAC across groups, W (2, 8.96) = 153.14, *p* < 0.001. TAC increased significantly in the AFG-L (9.44 ± 0.45; 5.73, 95% CI [4.28, 7.17], *p* < 0.001) and in the AFG-H groups (6.81 ± 0.16; 3.09, 95% CI [2.53, 3.66], *p* < 0.001) than the C group (3.71 ± 0.12). TAC significantly increased in the AFG-L group than the AFG-H group (2.63, 95% CI [1.19, 4.08], *p* = 0.003). Splenic SOD activity levels differed significantly across groups, W (2, 9.82) = 153.89, *p* < 0.001. SOD increased significantly in the AFG-L group (130.34 ± 2.08; 48.13, 95% CI [40.73, 55.53], *p* < 0.001) and in the AFG-H group (108.32 ± 0.16; 26.11, 95% CI [−18.08, 34.14], *p* < 0.001) than the C group (82.21 ± 1.69). SOD significantly increased in the AFG-L group than the AFG-H group (22.02, 95% CI [13.44, 30.60], *p* < 0.001). Splenic GPx activity levels differed significantly across groups, W (2, 9.58) = 142.44, *p* < 0.001. GPx increased significantly in the AFG-L group (132.88 ± 1.55; 50.95, 95% CI [42.54, 59.37], *p* < 0.001) and in the AFG-H group (111.08 ± 2.15; 29.15, 95% CI [20.02, 38.29], *p* < 0.001) than the C group (81.93 ± 1.69). GPx significantly increased in the AFG-L group than the AFG-H group (21.80, 95% CI [14.42, 29.18], *p* < 0.001). Splenic MDA levels differed significantly across groups, W (2, 9.57) = 39.45, *p* < 0.001. MDA decreased significantly in the AFG-L group (1.29 ± 0.03; 0.58, 95% CI [0.39, 0.77], *p* < 0.001) and in the AFG-H group (1.55 ± 0.04; 0.32, 95% CI [0.13, 0.51], *p* = 0.004) than the C group (1.87 ± 0.06). MDA significantly reduced in the AFG-L group than the AFG-H group (0.26, 95% CI [0.13, 0.39], *p* < 0.001). Splenic PCO levels differed significantly across groups, W (2, 9.17) = 234.43, *p* < 0.001. PCO levels decreased significantly in the AFG-L group (2.72 ± 0.13; 3.29, 95% CI [2.86, 3.73], *p* < 0.001) and in the AFG-H group (3.39 ± 0.19; 2.62, 95% CI [1.99, 3.26], *p* < 0.001) than the C group (6.01 ± 0.09). PCO levels significantly decreased in the AFG-L group than the AFG-H group (0.67, 95% CI [0.01, 1.33], *p* = 0.047). Splenic 8OH2dG levels differed significantly across groups, W (2, 9.69) = 147.22, *p* < 0.001. 8OH2dG decreased significantly in the AFG-L group (3.74 ± 0.18; 3.22, 95% CI [2.63, 3.80], *p* < 0.001) and in the AFG-H group (4.84 ± 0.12; 2.12, 95% CI [1.69, 2.55], *p* < 0.001) than the C group (6.96 ± 0.11). 8OH2dG levels significantly decreased in the AFG-L group than the AFG-H group (1.10, 95% CI [0.51, 1.69], *p* = 0.002).

### 3.6 Effect of *Achillea Fragrantissima* extract administration on testosterone synthesis pathway and immune-related genes in testes and spleen

**Figure 3:**
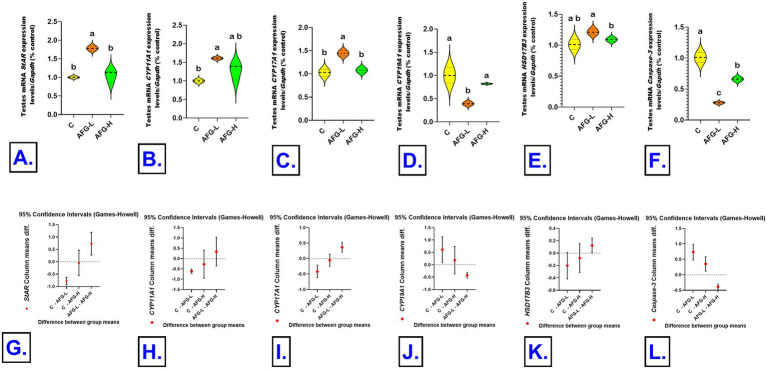
Effects of *A. fragrantissima* extract oral administration for 30 days on relative mRNA expression of steroidogenesis-related genes in testes of male Sprague Dawley rats. Group differences were analyzed using Welch’s ANOVA followed by Games–Howell multiple-comparison tests (*n* = 3 samples/group). Data are presented as violin plots showing distribution density with mean and interquartile range. Plots carrying different superscript letters indicate a significant difference between groups (*p* < 0.05). **(A)** Testicular *StAR*; **(B)** Testicular *CYP11A1*; **(C)** Testicular *CYP17A1*; **(D)** Testicular *CYP19A1*; **(E)** Testicular *HSD17B3*; **(F)** Testicular *Caspase-3*; and **(G–L)** Error bars represent pairwise comparisons and are reported with 95% confidence intervals (CI 95%) for the mean differences (*p* < 0.05).

**Figure 4:**
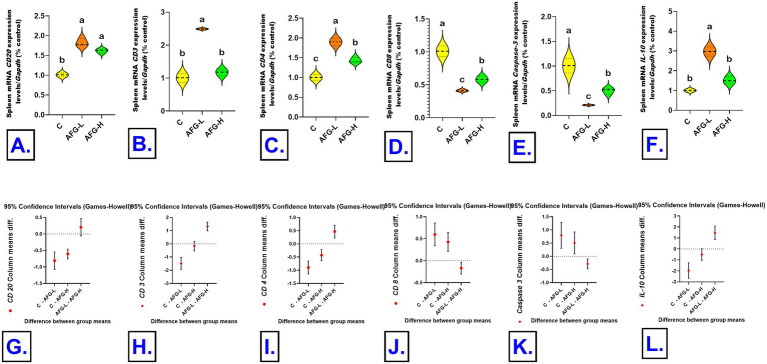
Effects of *A. fragrantissima* extract oral administration for 30 days on relative mRNA expression of immune-related genes in the spleen of male Sprague Dawley rats. Group differences were analyzed using Welch’s ANOVA followed by Games–Howell multiple-comparison tests (*n* = 3 samples/group). Data are presented as violin plots showing distribution density with mean and interquartile range. Plots carrying different superscript letters indicate a significant difference between groups (*p* < 0.05). **(A)** Spleen *CD20*; **(B)** Spleen *CD3*; **(C)** Spleen *CD4*; **(D)** Spleen *CD8*; **(E)** Spleen *Caspase-3*; and **(F)** Spleen *IL-10*, and **(G–L)** Error bars represent pairwise comparisons reported with 95% confidence intervals (CI 95%) for the mean differences (*p* < 0.05).

Figure 3 presents the effects of AFG administration on testicular levels of *StAR*, *CYP11A1*, *CYP17A1*, *CYP19A1*, *HSD17B3*, and *Caspase-3*. The mRNA expression levels of the *StAR* gene in testes differed significantly across groups, W (2, 3.22) = 193.94, *p* < 0.001. *StAR* expression levels were upregulated significantly in the AFG-L group (1.78 ± 0.032; 0.78, 95% CI [0.62, 0.93], *p* < 0.001) but, non-significantly in the AFG-H group (1.05 ± 0.091; 0.045, 95% CI [−0.47, 0.56], *p* = 0.885) than the C group (1.00 ± 0.016). *StAR* expression was significantly upregulated in the AFG-L group than the AFG-H group (0.73, 95% CI [0.27, 1.20], *p* = 0.017). The expression levels of the *CYP11A1* gene in testes differed significantly across groups, W (2, 3.40) = 161.17, *p* < 0.001. *CYP11A1* expression levels were upregulated significantly in the AFG-L group (1.61 ± 0.017; 0.60, 95% CI [0.49, 0.72], *p* < 0.001) but non-significantly in the AFG-H group (1.27 ± 0.12; 0.27, 95% CI [−0.40, 0.94], *p* = 0.270) than the C group (1.00 ± 0.026). No significant difference in the *CYP11A1* expression levels between the AFG-L and the AFG-H groups (0.34, 95% CI [−0.36, 1.03], *p* = 0.191). The expression levels of the *CYP17A1* gene in testes differed significantly across groups, W (2, 3.93) = 38.78, *p* = 0.003. *CYP17A1* expression levels were upregulated significantly in the AFG-L group (1.44 ± 0.032; 0.42, 95% CI [0.22, 0.61], *p* = 0.004) but non-significantly in the AFG-H group (1.08 ± 0.029; 0.05, 95% CI [−0.14, 0.25], *p* = 0.614) than the C group (1.00 ± 0.042). *CYP17A1* expression was significantly upregulated in the AFG-L group than the AFG-H group (0.36, 95% CI [0.21, 0.52], *p* = 0.003). The expression levels of the *CYP19A1* gene in testes differed significantly across groups, W (2, 2.77) = 193.87, *p* = 0.001. *CYP19A1* expression levels were downregulated significantly in the AFG-L group (0.394 ± 0.019; 0.61, 95% CI [0.07, 1.15], *p* = 0.039) but non-significantly in the AFG-H group (0.819 ± 0.004; 0.19, 95% CI [−0.39, 0.75], *p* = 0.334) than the C group (1.00 ± 0.097). *CYP19A1* expression was significantly downregulated in the AFG-L group than the AFG-H group (0.42, 95% CI [0.32, 0.53], *p* = 0.003). The expression levels of the *HSD17B3* gene in testes differed significantly across groups, W (2, 3.62) = 9.51, *p* = 0.036. No significant differences in *HSD17B3* expression levels between the AFG-L (1.21 ± 0.025; 0.21, 95% CI [−0.01, 0.43], *p* = 0.058) and the C group (1.00 ± 0.046) or between the AFG-H (1.09 ± 0.017; (0.08, 95% CI [−0.15, 0.32], *p* = 0.371) and the C group. *HSD17B3* expression was significantly upregulated in the AFG-L group than the AFG-H group (0.12, 95% CI [0.007, 0.24], *p* = 0.041). The expression levels of the *Caspase-3* gene in testes differed significantly across groups, W (2, 3.24) = 240.89, *p* < 0.001. *Caspase-3* expression levels were downregulated significantly in the AFG-L group (0.279 ± 0.008; 0.73, 95% CI [0.47, 0.98], *p* = 0.006) and in the AFG-H group (0.66 ± 0.017; 0.34, 95% CI [0.12, 0.57], *p* = 0.019) than in the C group (1.00 ± 0.045). *Caspase-3* expression was significantly downregulated in the AFG-L group than the AFG-H group (0.38, 95% CI [0.30, 0.47], *p* = 0.001). Figure 4 presents the effects of AFG administration on spleen mRNA expression levels of *CD20*, *CD3*, *CD4*, *CD8*, *IL-10*, and *Caspase-3*. The mRNA expression levels of *CD20* gene in spleen differed significantly across groups, W (2, 3.80) = 126.24, *p* < 0.001. *CD20* expression levels upregulated significantly in the AFG-L group (1.82 ± 0.055; 0.81, 95% CI [0.55, 1.07], *p* = 0.002) and in the AFG-H group (1.62 ± 0.028; 0.61, 95% CI [0.46, 0.75], *p* < 0.001) than the C group (1.00 ± 0.029). No significant differences in the *CD20* expression between the AFG-L and the AFG-H groups (0.20, 95% CI [−0.06, 0.46], *p* = 0.094). The mRNA expression levels of *CD3* gene in spleen differed significantly across groups, W (2, 2.86) = 345.61, *p* < 0.001. *CD3* expression levels upregulated significantly in the AFG-L group (2.48 ± 0.012; 1.48, 95% CI [1.05, 1.91], *p* = 0.004) but, non-significantly in the AFG-H group (1.18 ± 0.056; 0.17, 95% CI [−0.18, 0.52], *p* = 0.295) than the C group (1.00 ± 0.076). *CD3* expression significantly upregulated in the AFG-L than the AFG-H group (1.31, 95% CI [1.00, 1.62], *p* = 0.002). The mRNA expression levels of *CD4* gene in spleen differed significantly across groups, W (2, 3.95) = 74.76, *p* < 0.001. *CD4* expression levels upregulated significantly in the AFG-L group (1.90 ± 0.051; 0.90, 95% CI [0.65, 1.14], *p* < 0.001) and in the AFG-H group (1.43 ± 0.038; 0.43, 95% CI [0.21, 0.64], *p* = 0.005) than the C group (1.00 ± 0.045). *CD4* expression significantly upregulated in the AFG-L than the AFG-H group (0.47, 95% CI [0.24, 0.71], *p* = 0.005). The mRNA expression levels of *CD8* gene in spleen differed significantly across groups, W (2, 3.06) = 75.37, *p* = 0.002. *CD8* expression levels downregulated significantly in the AFG-L group (0.413 ± 0.009; 0.60, 95% CI [0.33, 0.86], *p* = 0.009) and in the AFG-H group (0.583 ± 0.025; 0.42, 95% CI [0.20, 0.64], *p* = 0.008) than the C group (1.00 ± 0.047). *CD8* expression significantly downregulated in the AFG-L than the AFG-H group (0.17, 95% CI [0.04, 0.30], *p* = 0.026). The mRNA expression levels of *Caspase-3* gene in spleen differed significantly across groups, W (2, 2.76) = 67.67, *p* = 0.005. *Caspase-3* expression levels downregulated significantly in the AFG-L group (0.208 ± 0.005; 0.80, 95% CI [0.32, 1.28], *p* = 0.019) and in the AFG-H group (0.498 ± 0.032; 0.51, 95% CI [0.10, 0.92], *p* = 0.030) than the C group (1.00 ± 0.082). *Caspae-3* expression significantly downregulated in the AFG-L than the AFG-H group (0.29, 95% CI [0.11, 0.47], *p* = 0.019). The mRNA expression levels of *IL-10* gene in spleen differed significantly across groups, W (2, 3.18) = 88.07, *p* = 0.002. *IL-10* expression levels upregulated significantly in the AFG-L group (2.97 ± 0.13; 1.97, 95% CI [1.27, 2.66], *p* = 0.005) but non-significantly in the AFG-H group (1.51 ± 0.104; 0.50, 95% CI [−0.017, 1.03], *p* = 0.054) than the C group (1.00 ± 0.039). *IL-10* expression significantly upregulated in the AFG-L than the AFG-H group (1.46, 95% CI [0.85, 2.08], *p* = 0.003).

### 3.7 Effects of *Achillea Fragrantissima* extract administration on testes and spleen histopathological investigation

**Figure 5:**
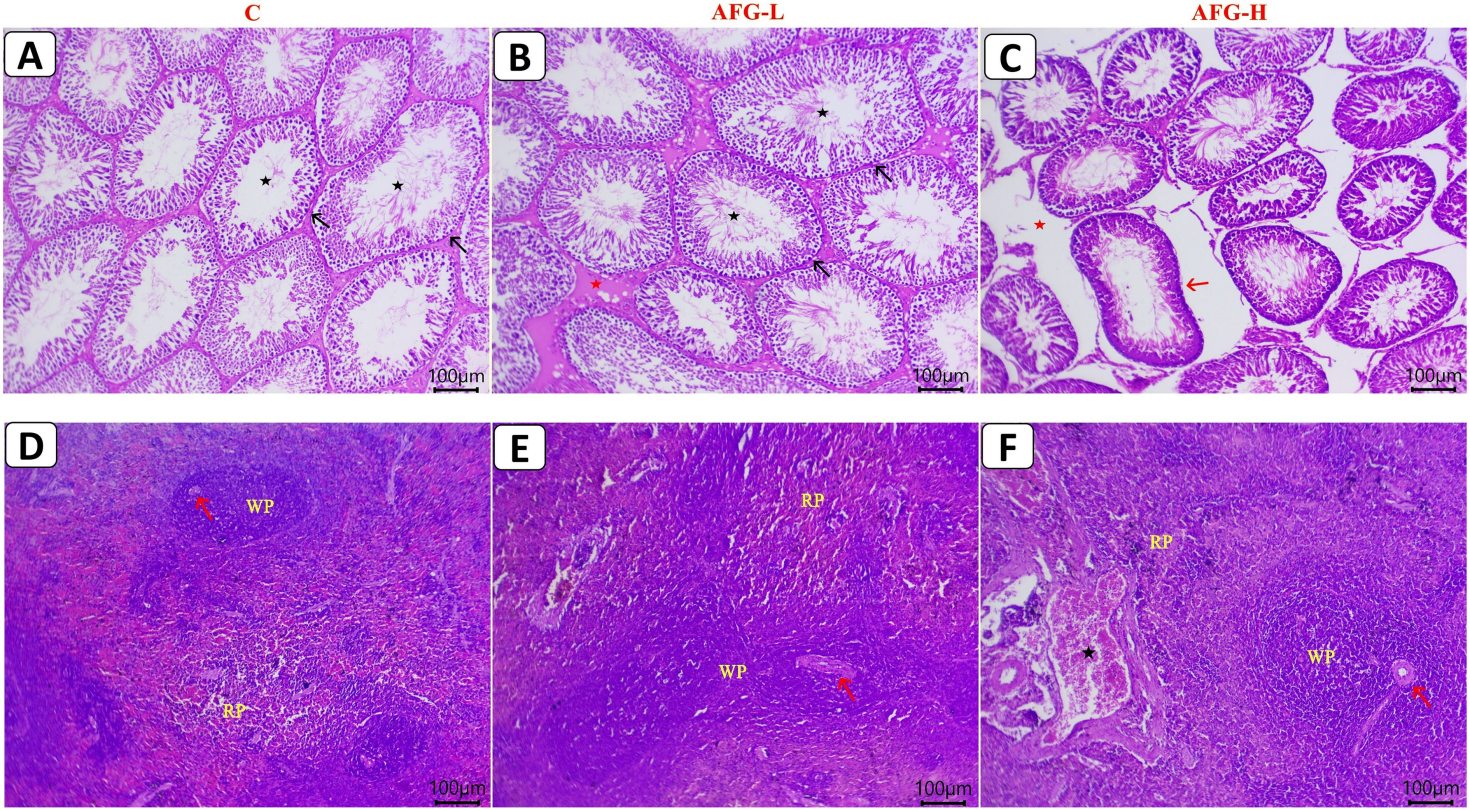
Photomicrographs of H&E-stained tissue sections of testes (scale bar 100 μm) showing **(A)** normal histology of seminiferous tubules (black arrows) with intraluminal mature sperms (black stars) and normal interstitial tissues at control group, **(B)** apparent normal seminiferous tubules (black arrows) with mature sperms (black stars) with mild interstitial edema (red star) in the AFG-L group, **(C)** irregularity of some seminiferous tubules (red arrow) with less numbers of intraluminal mature sperms beside interstitial edema between tubules (red star) in the AFG-H group, **(D)** normal histological architectures of white pulp lymphoid follicles around eccentric arterioles and typical red pulps at control group, **(E)** normal histological architectures of white pulp lymphoid follicles around eccentric arterioles and typical red pulps at AFG-L group, and **(F)** preserved structures of lymphoid follicles at white pulps beside congested vasculatures (black star) at red pulp at AFG-H group (C). White pulp (WP), red pulp (RP), eccentric arterioles (red arrows). The frequencies of the encountered morphological changes in all groups were presented as (means ± SEM) of 10 animals per experimental grouping.

The examined testicular section of the 1st group of rats (C) showed normal histology of seminiferous tubules and interstitial tissues. The seminiferous tubules were lined by spermatogonia, spermatocytes, spermatids, centrally located mature sperms, and Sertoli cells (Figure 5A). Moreover, the 2nd rat group (AFG-L) showed apparently normal morphological structures of seminiferous tubules with non-significant mild interstitial edema (Figure 5B). Furthermore, the examined testicular sections of the 3rd group of rats (AFG-H) exhibited irregularity of some seminiferous tubules with fewer numbers of intraluminal mature sperms, besides interstitial edema between tubules (Figure 5C). All observed morphological changes were non-significant between the control and the low-dose-administered experimental rat groupings. Meanwhile, the examined spleen sections of the control rat group (C) displayed normal histological architectures of white pulp lymphoid follicles around eccentric arterioles and normal red pulp (Figure 5D). The red pulps were involved dilated sinusoids, erythrocytes, lymphocytes, reticular fibers, and many macrophages in the spleen sections of the AFG-L group (Figure 5E). Moreover, spleen sections of the AFG-H group showed preserved structures of lymphoid follicles at white pulps beside congested vasculatures at red pulp (Figure 5F). The frequencies of the encountered morphological changes in all groups were presented as (means ± SEM) and summarized in Table 6.

**Table 6:** Effect of *A. fragrantissima* extract oral administration on testes and spleen histopathology of adult male Sprague Dawley rats for 30 days.

Tissue examined	Lesion type	1st control	2nd AFG-L	3rd AFG-H	Mean Rank (G1/G2/G3)	H (2)	*p*-value
Testicular tissue histopathological lesions scoring	Germinal epithelium atrophy	0.00 ± 0.00 ^b^	3.00 ± 1.53 ^b^	12.00 ± 2.00 ^a^	9.50/13.55/23.45	17.56	*p* < 0.001
	Germinal epithelium apoptosis	0.00 ± 0.00 ^b^	3.00 ± 1.53 ^b^	12.00 ± 2.00 ^a^	9.50/13.55/23.45	17.56	*p* < 0.001
	Germinal epithelium vacuolation	0.00 ± 0.00 ^b^	6.00 ± 1.63 ^ab^	13.00 ± 2.13 ^a^	8.00/15.80/22.70	16.94	*p* < 0.001
	Interstitial space with exudates	0.00 ± 0.00 ^b^	9.00 ± 2.76 ^ab^	18.00 ± 3.26 ^a^	8.00/16.10/22.40	15.66	*p* < 0.001
	Interstitial space with inflammatory cell infiltrations	0.00 ± 0.00 ^b^	6.00 ± 1.63 ^ab^	12.00 ± 2.00 ^a^	8.00/16.10/22.40	16.58	*p* < 0.001
	Interstitial congestion	0.00 ± 0.00 ^b^	6.00 ± 1.63 ^ab^	12.00 ± 2.00 ^a^	8.00/16.10/22.40	16.58	*p* < 0.001
	Basement membrane irregularities	0.00 ± 0.00 ^b^	3.00 ± 1.53 ^b^	24.00 ± 3.05 ^a^	9.50/12.65/24.35	20.39	*p* < 0.001
Spleen tissue histopathological lesions scoring	Lymphoid depletion	0.00 ± 0.00 ^a^	0.00 ± 0.00 ^a^	3.00 ± 1.52 ^a^	14.00/14.00/18.50	6.44	*p* = 0.040
	Lymphoid necrosis	0.00 ± 0.00 ^a^	0.00 ± 0.00 ^a^	3.00 ± 1.52 ^a^	14.00/14.00/18.50	6.44	*p* = 0.040
	Endothelial hypertrophy	0.00 ± 0.00 ^b^	3.00 ± 1.53 ^b^	12.00 ± 2.00 ^a^	9.50/13.55/23.45	17.56	*p* < 0.001
	Vascular congestion	0.00 ± 0.00 ^b^	6.00 ± 1.63 ^b^	24.00 ± 3.05 ^a^	8.00/14.30/24.20	20.04	*p* < 0.001
	Hemosiderosis	0.00 ± 0.00 ^b^	6.00 ± 1.63 ^b^	24.00 ± 3.05 ^a^	8.00/14.30/24.20	20.04	*p* < 0.001
	Vacuolation	0.00 ± 0.00 ^b^	3.00 ± 1.53 ^b^	12.00 ± 2.00 ^a^	9.50/13.55/23.45	17.56	*p* < 0.001

Values are mean ± SEM of 10 animals per experimental group. Kruskal-Wallis test followed by Dunn’s multiple comparisons test. Groups carrying the same superscript letters indicate no significant differences at the *p* < 0.05 level.

## 4 Discussion

Medicinal plants encompass a diverse array of phytochemicals, such as flavonoids, phenolic compounds, quinones, triterpenoids, alkaloids, steroids, and terpenoids, which may be beneficial in addressing fertility-related concerns (13). Therefore, it is essential to investigate the male fertility potential of medicinal plants for the significant management of various health disorders, including fertility-related difficulties (54). *Achillea* spp. shows promise as a potential source for pharmacological compounds that target different disorders. *Achillea fragrantissima* has been traditionally consumed to treat several diseases and is considered a plant of phyto-pharmaceutical significance, containing a wide range of phytochemicals including total phenols, flavonoids, tannins, and alkaloids (55). The obtained findings in the current investigation revealed that AFG extract at both high and low doses modulated the behavioral observations of exposed male rats. The behavioral tests were chosen to assess physiological and neurobehavioral aspects that influence male reproductive function, either directly or indirectly, through CNS regulation of the hypothalamic–pituitary–gonadal (HPG) axis, sexual motivation, and mating competence.

Results of the current study showed that AFG enhanced feeding and drinking behaviors in male rat which are indicators of general metabolic status and hypothalamic function, both of which are tightly linked to reproductive endocrinology. Alterations in energy balance or hydration can disrupt gonadotropin secretion and testosterone production, thereby indirectly affecting spermatogenesis and sperm quality. Regarding the results of the rat tube dominance test, administration of AFG extract significantly augmented forward walking (FW) and reduced backward walking (BW) in the AFG-L group compared to the C and the AFG-H groups. The tube dominance test was utilized to assess social dominance and competitive behavior. Social dominance and aggression are hormonally regulated by androgens and estrogens, which significantly affect mating access, reproductive investment, and overall fitness. Dominance hierarchies influence reproductive possibilities in numerous species. Dominant rats typically exhibit increased FW time and decreased BW time, reflecting a proactive “winner’s mentality” and a proactive inclination to expel the subordinate rat from the tube. It was reported that *Achillea* species, particularly *A. millefolium* and *A. wilhelmsii*, contain bioactive compounds with anxiolytic properties that may affect behavior linked to dominance. Studies have shown that *A. millefolium’s* hydroalcoholic extract possesses anxiolytic-like effects, demonstrated in multiple animal assays, partially through the GABA/benzodiazepine system (56). Additionally, *A. wilhelmsii’s* essential oil demonstrated anxiolytic properties in behavioral assessments (57). The polar fraction of *A. millefolium* extract was more effective in sedative and anxiolytic effects compared to other fractions (58). Inhalation of *A. biebersteinii* essential oil reduced anxiety and depressive behaviors in amnesic rats (59). However, the specific effects of *A. fragrantissima* extracts on the tube dominance test remain unexplored. This is the first study to report the impact of *A. fragrantissima* extracts on the tube dominance test.

Regarding the effect of *A. fragrantissima* on Y-Maze observations, the *A. fragrantissima* extracts improved the spontaneous alternation percentage (SAP) in both AFG-L and AFG-H groups relative to the C rats. The Y-maze test is frequently used to assess experimental animals’ short-term memory, based on rodents’ natural tendency to explore unfamiliar environments. Discovery behavior and an interest in odd locations and things have long been linked to spatial memory. Animals attempt to learn about new surroundings when they are presented with them (60). Cognitive and motivational mechanisms influence sexual behavior and partner recognition, and are responsive to hormonal and oxidative alterations that impact reproductive physiology. Hormonal changes during the cycle influence behavioral performance in cognitive activities. Cognitive assays provide valuable insights for evaluating hormone-dependent neural integrity. Several species of *Achillea* have demonstrated encouraging benefits on memory and cognitive abilities, particularly with regard to the Y-maze test, which assesses mice’s spatial working memory. Inhalation of *A. pseudoaleppica* essential oil markedly enhanced memory in scopolamine-induced amnesic rats. The essential oil augmented spontaneous alternation behavior in the Y-maze test, signifying improved spatial working memory. The essential oil reduced acetylcholinesterase activity and OS markers in the hippocampus, hence boosting memory through its antioxidant and anticholinesterase properties (61). Also, *A. millefolium* has demonstrated potential in enhancing cognitive abilities. The hydroalcoholic extract of this species had anxiolytic-like effects in multiple behavioral assessments, perhaps enhancing cognitive performance. Extract of *A. fragrantissima* may enhance GABAergic inhibition in the CNS.

The phytochemical ingredients might contribute to *A. fragrantissima*’s properties. CNS depressant and anxiolytic activity of *A. fragrantissima* was supposed to be attributed to these phytochemicals found in the extract. That is in agreement with El-Ashmawy et al. (33); Baretta et al. (56); Elmann et al. (62); and Tarawneh et al. (63). Antioxidant supplementation may enhance cognitive performance in various species, including rodents (64), humans (65), and dogs (66). Antioxidants also demonstrated their efficacy in alleviating anxiety-related behaviors in mice and rats (67). Nevertheless, there are existing studies that have accounted for specific oral administration of *A. fragrantissima* in relation to oxidative stress and subsequent learning performance or anxiety-related behaviors. Thus, this study was designed to evaluate the impacts of *A. fragrantissima* oral administration on behaviors in rats. Flavonoids have been identified to bind with the CNS’s GABAA receptors, suggesting that flavonoids have benzodiazepine effects, which is corroborated by flavonoid-related behavior impacts in anxiety, sedation, and convulsions induced animal models (68). According to previous studies, chlorogenic acid is regarded as a neuroactive compound, as it can improve cognitive function as evaluated by tests of passive avoidance and Y-maze, both of which are measures of working memory deficits and short-term memory impaired by scopolamine (69). Similarly, the hydro alcoholic extract of *A. wilhelmsii* elevated nitric oxide metabolite levels in the hippocampus and influenced the severity of a seizure model induced by pentylenetetrazole (70). Furthermore, monoterpenes’ ability to improve memory has been documented previously (71). The effects of *A. pseudoaleppica* essential oil on memory may be connected to its effects on oxidative stress. Numerous *Achillea* extracts and essential oils have been shown in the past to reduce OS in animal brains (72).

Grooming has been characterized as behavior linked to heightened anxiety (73). In our investigation, grooming frequency diminished as the dosage of AFG increased. The rationale for grooming has been contested, with some linking it to anxiety and stress (74), while others perceive it as a “de-arousal” mechanism that transpires following a stressful event (75). This discourse centers on the temporal frequency of behavior. A decrease in behavior with repeated exposure is considered a stress-related response during habituation (76). A behavior that escalates over time may contribute to de-arousal following the cessation of stress (75). In our investigation, grooming diminished, corroborating the stress hypothesis. Rats subjected to the AFG demonstrated significantly reduced grooming behavior compared to control rats, potentially indicating lower stress levels (77). Frequency and duration of grooming in our study decreased as the dose of *A. fragrantissima* increased. Grooming, as a frequent behavior, appears frequently in humans and resembles self-grooming behaviors in models of animals (78). Moreover, self-grooming is identified as a behavioral indicator of stress and anxiety, since rodents frequently engage in excessive grooming when confronted with stressful stimuli (79). The hole-board test was employed to evaluate exploration, locomotion, and anxiety-related reactions. Anxiety and stress are recognized to inhibit reproductive function by activating the hypothalamic–pituitary–adrenal axis, which adversely affects the hypothalamic–pituitary–gonadal axis. Exploration and anxiety are influenced by sex hormones; the estrus stage affects behavior in tests such as the hole-board test. These behaviors affect partner selection, territoriality, and reproductive social interactions.

Swimming performance was utilized as an indicator of physical endurance and neuromuscular coordination, both of which are pertinent to mating competence and overall vitality. Decreased physical performance may obscure reproductive outcomes by restricting sexual activity instead of indicating direct reproductive harm. Stress hormones also modify reproductive physiology, rendering stress-related performance related to fertility outcomes. Concerning the impact of AFG extract on male reproductive performance, the findings displayed that semen parameters and hormonal levels of TEST, LH, FSH, and E2, as well as the testes histological structure, were enhanced more markedly in the AFG-L group than the AFG-H one. Similar findings were obtained by Mandour et al. (80), who reported that neither the aqueous, methanolic, nor ethanolic extract at 250 mg/kg altered the male fertility or hematological parameters in the treated rats for 2 weeks, 1 month, or 2 months. This is the study to report the effect of *A. fragrantissima* on male sexual hormones and OS in testicular tissue. Such effects may be attributed to its potent antioxidant properties owing to its high content of phenolics and flavonoids (18, 19, 33, 81–83). Similar results obtained with other *Achillea* species, like *A. millefolium* 1.2 g/kg orally for 48 days enhanced sperm count, motility, viability, FSH, LH, TEST, and antioxidant capacity but decreased abnormal sperms in testes of nicotine exposed rats (84), as well as Salahipour et al. (25) reported that *A. millefolium* inflorescences alcoholic extract protected against male reproductive failures brought on by nicotine exposure. In the present study, the serum E2 levels did not decrease alongside the downregulated expression of testicular *CYP19A1*. This could be attributed to the fact that about 80% of circulating E2 in males is released from extra gonadal tissues, with only a minor 15% produced by the testes (85, 86); thus, testicular *CYP19A1* downregulation has little impact on serum E2 levels. Several mechanisms clarified these findings: firstly, the extra gonadal aromatization serves as a primary compensatory source of circulating estrogen in male mammals, including rats (86, 87). Consequently, E2 production from extra gonadal tissues such as the brain, liver, adipose tissue, bone, skin, muscles, vasculature, and adrenal sources may compensate for the decreased testicular *CYP19A1* activity. Previous studies showed that serum E2 levels do not consistently reduce when testicular *CYP19A1* is downregulated; for instance, vitamin D3 can increase testicular *CYP19A1* expression without affecting serum E2 levels (88), pointing to independent regulation of aromatase in extra-gonadal tissues. Moreover, changes in the aromatase enzyme activity may arise from post-transcriptional and post-translational regulation of *CYP19A1* that are not apparent at the mRNA level. For example, in rare minnow males exposed to chlordecone, testicular *CYP19A1* mRNA was markedly downregulated, although serum levels of E2 and the E2/TEST ratio were elevated, suggesting that under specific endocrine-disrupting conditions, circulating E2 may increase in males despite reduced testicular aromatase gene expression (89). Lastly, changes in endocrine feedback mechanism within the hypothalamic–pituitary-gonadal (HPG) axis can modify systemic hormone levels without directly affecting molecular expressions. Despite reduction in testicular *CYP19A1* expression, hormonal feedback has improved the increase in circulating E2 levels. This disruption in steroid homeostasis may trigger a compensatory auto-regulation, leading to decreased transcription of E2 synthesis enzymes (*CYP19A*) and increased transcription of steroidogenic enzymes involved in androgen synthesis (*CYP11A* and *CYP17*). Accordingly, the observed increase in circulating E2 and TEST in the current study was likely due to enhanced steroidogenesis driven by the upregulated transcription of *CYP11A* and *CYP17*, resulting in a negative feedback loop within the HPG axis, which subsequently downregulated brain Gnrh3 and *CYP19B* mRNA levels (89). *CYP19A1* transcription responds differently in each tissue due to multiple promoters, enabling tissue-specific compensation (85). Some research only infers processes based on changes in gene expression, and not all studies actually detect both *CYP19A1* expression and serum E2, which is considered a limitation. Nevertheless, despite male testicular *CYP19A1* mRNA being downregulated, the evidence suggests that there may be elevated blood E2, mostly as a result of extra-gonadal production and endocrine disturbance (89). Regarding the effect on immune response, AFG extract significantly elevated Ph% and PhI. Similar positive effects of *A. fragrantissima* oil on cellular and humoral immune response were reported in mice injected intraperitoneally with 10, 20, and 40 mg/kg B.wt. AFG oil twice daily for 10 days significantly improved the hemagglutination index in the three-dose group relative to the C animals, which was accompanied by a decrease in the swelling differential in the feet and an increase in spleen weight (17). *Achillea fragrantissima* extract at 500 mg/kg B.wt., for 38 days resulted in a significant hypertrophy of white pulp and hyperplasia of lymphoid cells, with microscopic immune reactions scores indicating moderate to high immunostimulation levels, suggesting that *A. fragrantissima* may enhance immune response which might be attributed to its antioxidative characteristics and phytoconstituents (Camphor, 1, 8-Cineole, Artemisia ketone, Thujone, and Cyclohexene, 3-(1, 5-dimethyl-4-hexenyl)-6-methylene) identified. The antioxidants in AFG appear to safeguard the WBC involved in immune responses against OS, hence inhibiting the death of such cells (90).

Regarding the anti-inflammatory effect of AFG, findings exhibited that a low dose of AFG decreased the serum levels of IL-6 and TNF-*α* inflammatory cytokines, as well as the testicular LDH levels. Similar results were reported by Alhomaid et al. (17), where AFG extract at 300 mg/kg decreased TNF-α but significantly elevated IL-4 and IL-10. Similarly, higher dose levels at 500 mg/kg significantly modulated IL-10. The results align with several studies concerning other *Achillea* species (91). Limited research has been conducted on AFG that might substantially decrease proinflammatory cytokines, indicating its potential cyto-protective impact against cytokine imbalances (19). Administration of AFG ethanol or ethyl acetate extracts normalizes the inflammation-related cytokines in the serum owing to its anti-inflammatory properties (92). AFG extract primarily inhibited TNF-α expression and downregulated ROS generation induced by lipopolysaccharide (23). AFG extract may prevent the activation of Nuclear Factor Kappa B, thus triggering pro-inflammatory protein kinase C or p38 MAPK production (93).

Regarding *CD4* expression, Sprague–Dawley rats given *A. wilhelmsii* methanolic extract at 75 mg/kg daily over 1 month significantly decreased TGs and VLDL, and increased *CD4* expression in lymphocytes and monocytes, supporting that *A. wilhelmsii* displayed immunomodulatory consequences via induction of *CD4* expression on T helper lymphocytes. The concentrations of IL-1β, IL-6, IL-10, and TNF-α displayed no induction of inflammation or hepatic injury (94).

The current study findings suggest a biphasic modulatory effect of AFG extract administration on the immune response. Results indicate a dose-dependent modulation characterized by adaptive immune enhancement at low dose but proinflammatory dysregulation at high dose. Low doses improve innate immunity by increasing Ph% and PhI while reducing TNF-α and IL-6 cytokines, reflecting an anti-inflammatory profile. Upregulation of splenic *CD20*, *CD3*, *CD4*, and *IL-10* suggests immune cell activation and better immunological balance, with reduced apoptosis marked by lower *CD8* and *Caspase-3* levels. However, the AFG-H group showed elevated TNF-α and IL-6 levels, decreased granulocyte %, and reduced phagocytic activity, indicating an inflammatory response. This suggests that while low doses foster beneficial immune responses, high doses may disrupt immune homeostasis and provoke inflammation. Regarding OS-related indices, our findings showed that AFG extract significantly increased SOD, GPx, and TAC in testes and spleen, but decreased levels are similar to those of Alhomaid et al. (17). Additionally, Hijazi et al. (19) reported that oral *A. fragrantissima* extract considerably reduced the adriamycin-induced increase in TBARS levels in heart tissue. GPx and GSH were markedly increased in heart tissue. As well, the administration of *A. fragrantissima* methanolic extract diminished LPO and elevated the reduced GSH levels (33)*. Achillea fragrantissima* supplementation considerably mitigated the detrimental effects of endoxan via a notable elevation in serum SOD, modulations of CAT, TAC, and MDA contingent upon the dosage. The administration of ethanol extract from AFG resulted in a considerable reduction in MDA levels (92). The SOD serves as the primary defensive mechanism against ROS. It facilitates the conversion of two superoxide molecules into molecular oxygen and hydrogen peroxide, hence rendering superoxide radical less detrimental.

*Achillea fragrantissima* has a powerful antioxidant effect, owing to its components with significant antioxidant activity, notably flavonoids and polyphenolics. Achillolide A, a sesquiterpene lactone in *A. fragrantissima*, reduced the increased levels of nitric oxide and ROS (95). *Achillea fragrantissima* biologically active compounds combat OS via ROS scavenging plus inhibiting the hydrogen peroxide-activated MAPK pathway. Sesquiterpene lactone Achilloid A mitigates H_2_O_2_-induced astrocytes by ROS scavenging and inhibiting the mitogen-activated protein kinase pathway triggered by H_2_O_2_ (96).

Flavonoids are phytocomponents that demonstrate antioxidant properties. A prior investigation found that flavonoid 3, 5, 4′-trihydroxy-6, 7, 3′-trimethoxyflavone (TTF), found in *A. fragrantissima*, mitigated H_2_O_2_-initiated astrocyte mortality and preserved the MAPK family cell-signaling proteins phosphorylation. TTF mitigates free radicals and reduces intracellular buildup of ROS after cells are exposed to H_2_O_2_ or to peroxyl radical-producing compound 2, 2′-azobis (amidinopropane; ABAP) (97). TTF, a natural flavonoid derived from *A. fragrantissima*, shows the ability to safeguard neuronal cells from Amyloid beta Aβ-induced cytotoxicity, inhibiting the activation of MAP kinases and reducing the ROS buildup inside cells after Aβ protein treatment (82).

The investigation demonstrated that the *A. fragrantissima* extract has high flavonoid and phenolic concentrations. In addition, Achilloid hindered the activation of Neuro2a cells’ stress-activated protein kinase/c-Jun N-terminal kinase and p44/42 mitogen-activated protein kinase, as well as decreased ROS buildup by Aβ, improved their viability, and lowered Aβ-induced mortality (81). Finally, *A. fragrantissima* methanolic extract has a significant phenolic and flavonoid concentration and showed robust antioxidative potential via scavenging diphenylpicrylhydrazyl (DPPH) free radicals (98). The phenolic compounds significantly scavenge ROS due to their reactivity as donors of hydrogen or electrons and their ability to chelate metal ions (99, 100).

Regarding the effect of *A. fragrantissima* on transcriptional levels of steroid hormone synthesis pathway-related genes, this is the first *in vivo* investigation that reports the impact of AFG-extract on gene expression in testes and immune-related genes in the spleen of AFG-administered rats. Results showed that AFG low dose significantly upregulated transcriptional levels of *StAR*, *CYP11A1*, *CYP17A1*, and *HSD17B3*; meanwhile, levels of *CYP19A1* and *Caspase-3* were significantly downregulated. Likely, the transcriptional levels of *CD20*, *CD3*, *CD4*, and *IL-10* were considerably upregulated in the spleen of the AFG-L group; meanwhile, levels of CD8 and Caspase-3 were significantly downregulated. On the contrary, the high dose level of AFG extract non-significantly modulated the expression levels of the previously mentioned genes. According to Acevedo-Rodriguez et al. (101), the HPG-axis serves as the primary fertility-controlling network, reproductive hormone production, and general reproductive function in animals. This axis controls the production and release of sex hormones by means of a synchronized process between the gonads, pituitary gland, and hypothalamus. Significant reproductive deficiencies can result from disruption at any point along the HPG axis (101, 102). Gonadotropin-releasing hormone (GnRH), is formed by the brain and controls the release of pituitary gonadotropins, such as FSH and LH. Mammalian reproductive physiology is largely regulated by GnRH (103). The testes’ Leydig cells need LH to stimulate testosterone synthesis, and a drop in LH levels is positively connected with less testosterone being produced. When cholesterol enters Leydig cells through the scavenger receptor class B member 1 (*SCARB1*), testosterone production starts. The steroidogenic acute regulatory protein (*StAR*) subsequently carries cholesterol to the inner mitochondrial membrane, where *CYP11A1* transforms it into pregnenolone. The smooth endoplasmic reticulum of Leydig cells contains the enzymes *CYP17A1*, 3β-hydroxysteroid dehydrogenase (*HSD3B1*), and 17β-hydroxysteroid dehydrogenase 3 (*HSD17B3*), which are involved in the subsequent enzymatic steps that synthesize testosterone (104).

The observed improvement in sperm quality following AFG administration appears to be mechanistically linked to enhanced antioxidant defense and activation of steroidogenic pathways. Testicular tissue is highly susceptible to OS due to its elevated metabolic activity and abundance of polyunsaturated fatty acids in germ cell membranes. Excessive ROS can damage sperm DNA, proteins, and LPO, resulting in impaired Leydig cell steroidogenesis, disruption of spermatogenesis supported by Sertoli cells (105–112). In the present study, AFG administration in the AFG-L group significantly increased testicular TAC and enzymatic antioxidants GPx and SOD, while concomitantly reducing OS-related markers, MDA, PCO, and 8OH2dG, which created a favorable microenvironment for normal testicular function. The reduction in OS by antioxidant supplementation may directly contribute to the observed upregulation of key steroidogenic genes, including *StAR*, *CYP11A1*, *CYP17A1*, and *HSD17B3*. ROS are known to downregulated *StAR* expression and mitochondrial cholesterol transport, thereby limiting testosterone biosynthesis. By mitigating oxidative damage, AFG may preserve mitochondrial integrity and enhance cholesterol transfer into mitochondria, leading to increased testosterone synthesis, restoration of FSH and LH levels, improvement of testicular histology, and reduction of germ cell apoptosis (105–112). Improved steroidogenic activity, together with reduced oxidative insult, provides a mechanistic basis for the enhanced sperm parameters induced by the AFG, including increased sperm count, motility, and viability, along with reduced sperm abnormalities and dead sperm %. TEST is critical for maintaining the integrity of the seminiferous epithelium, promoting germ cell differentiation, and regulating epididymal sperm maturation. Moreover, reduced oxidative damage to sperm membranes and DNA likely contributed to improved sperm functional quality. Collectively, these findings suggest that AFG exerts its beneficial effects on male reproductive performance through an integrated mechanism involving antioxidant protection, activation of steroidogenic gene expression, and preservation of spermatogenic efficiency.

Our study had some limitations that must be acknowledged when evaluating the findings. While our study analyzed multiple reproductive performance-related indices and gene expressions, additional research, including expression of *CYP19A1* in extra-gonadal tissues like the brain are essential to clarify the main potential mechanisms by which phytoconstituents found in *A. fragrantissima* account for enhancing reproductive performance. The origin of the plant material has been elucidated in the Methods section, specifying that it was acquired from a local market. Despite the taxonomic authentication of the plant material by a certified botanist, the lack of wild collection with GPS coordinates has been acknowledged as a shortcoming of the study. Another limitation is the absence of molecular docking analyses to evaluate the potential interactions between the major bioactive compounds identified in the extract and the key upregulated genes/proteins in testicular and splenic tissues. Future investigations will include these computational studies, which could provide deeper mechanistic insights into the obtained outcomes. The current data confirm our initial hypothesis that the oral administration of Achillea fragrantissima influences male reproductive performance via antioxidant, immunomodulatory, and steroidogenic processes. The low dose (500 mg/kg) specifically enhanced antioxidant defenses (elevated SOD, GPx, and TAC), diminished oxidative stress indicators (MDA, PCO, and 8OH2dG), and improved testicular histoarchitectures. The biochemical enhancements were associated with the activation of critical steroidogenic genes (*StAR*, *CYP11A1*, *CYP17A1*, and *HSD17B3*) and the downregulation of *CYP19A1* and *Caspase-3*, signifying increased testosterone production and less germ cell apoptosis. These molecular and biochemical alterations resulted in enhanced sperm count, motility, viability, and hormonal equilibrium (TEST, LH, and FSH), hence facilitating better spermatogenic efficiency. The enhancement of immunological markers (elevated Ph%, PhI, *CD20*, *CD3*, *CD4*, and *IL-10*; diminished *CD8* and *Caspase-3*) indicates that AFG at a low dosage promotes immune homeostasis while inhibiting inflammatory and apoptotic signals. The high dose (1,000 mg/kg) exhibited a biphasic response, characterized by diminished immunological equilibrium and a less significant increase in reproductive function, indicating dose-dependent regulation. The data collectively validate our hypothesis that AFG enhances male reproductive function chiefly through antioxidant-mediated preservation of testicular tissue, activation of steroidogenic pathways within the HPG axis, and maintenance of immunoregulatory balance.

## 5 Conclusion

The outcomes of the current study indicate that oral administration of *Achillea fragrantissima* extract produces a dose-dependent improvement in behavioral outcomes, male reproductive performance, antioxidant capacity, immune function, and mRNA regulation of essential steroidogenic genes in the testes, as well as immune-related genes in the spleen. The findings demonstrate that AFG at 500 mg/kg yielded more significant positive effects than the greater dosage of 1,000 mg/kg, as indicated by substantial increases in SOD, GPx, and TAC levels, alongside notable reductions in LDH, MDA, PCO, and 8OH2dG levels in both testicular and splenic tissues. The enhancements correlated with beneficial biochemical alterations in TEST, LH, FSH levels, and improved semen characteristics, and molecular modification marked by the upregulation of *StAR*, *CYP11A1*, *CYP17A1*, *CD20*, *CD3*, *CD4*, and *IL-10* expression; meanwhile, CYP19A1, HSD17B3, Caspase-3, and CD8 were downregulated. The advantageous benefits of *A. fragrantissima* are likely facilitated mainly by the bioactive phytochemicals found in its aerial components, especially phenolics and flavonoids, which augment antioxidant capacity and improve steroidogenic and immunomodulatory pathways.

## Data Availability

The datasets generated for this study are available on request to the corresponding authors.
